# Market potential for circular food in Europe based on consumer demand and willingness to pay

**DOI:** 10.1038/s41538-025-00475-y

**Published:** 2025-07-21

**Authors:** Shanshan Li, Zein Kallas, Selene Ivette Ornelas Herrera, Muhammad Adzran Che Mustapa, Lena Behrendt, Zoltán Hajdu, Evi Michels, Erik Meers

**Affiliations:** 1 School of Humanities and Law, Henan Agricultural University, Zhengzhou, China; 2 Institute for Research in Sustainability Science and Technology (IS-UPC), Universitat Politècnica de Catalunya (UPC), Barcelona, Spain; 3 Centre for Agro-Food Economics and Development-UPC-IRTA (CREDA), Universitat Politècnica de Catalunya (UPC), Castelldefels, Spain; 4 Department of Agrifood Engineering and Biotechnology (DEAB), Universitat Politècnica de Catalunya (UPC), Castelldefels, Spain; 5 THU, Thünen Institute of Farm Economics, Braunschweig, Germany; 6 SOLTUB, Soltub Trade and Service Providing Limited Liability, Budapest, Hungary; 7 Department of Green Chemistry and Technology, Ghent University, Ghent, Belgium

**Keywords:** Agriculture, Economics

## Abstract

Circular farming is a sustainable agricultural system that promotes waste reuse and nutrient recovery by minimizing external inputs, closing nutrient loops, and reducing environmental impact. To our knowledge, this study is the first to apply open-ended choice experiments (OECEs) to assess consumers’ willingness to pay (WTP) and demand for food products from circular farming, involving 5289 participants across six EU countries. Three product categories were analyzed: pork, milk, and bread. Results showed that there is market potential for the food products produced from circular farming in Europe, as consumers are willing to pay a premium for circular products compared to conventional ones. Spanish consumers exhibited the highest WTP premiums (>26%) across all categories, whereas Hungarian consumers showed the lowest premiums. Socio-demographic factors, environmental attitudes, and price levels significantly influenced the quantity of circular food products that consumers were willing to purchase.

## Introduction

Current intensive agricultural systems not only provide feed and food, but also make a major contribution to greenhouse gas emissions^1,2^. The expansion of intensive production related to animal husbandry has, directly and indirectly, led to air and water pollution and poses a major threat to the environment^3^. Furthermore, conventional agriculture largely follows a linear economy model (an open-loop system) of “take-make-dispose”, which involves taking resources from nature, making them into goods, and disposing of goods that humans do not need^4^. This model is based on the intensive consumption of natural resources, which damages agroecosystems and the environment^5^ and is proving to be environmentally unsustainable^6^. Additionally, with the world population expected to reach 9.7 billion by 2050, agriculture will be under enormous pressure^7^. Therefore, to alleviate the pressure arising from future population growth, it is important to improve soil quality, reduce greenhouse gas emissions, and increase agricultural sustainability and future output capacity. In this context, the emergence of the circular economy concept (a closed-loop system) and its application to agriculture is essential to reduce the negative impacts of conventional agricultural systems. The circular economy model helps to achieve sustainability by preventing environmental degradation and ensuring the economic and social well-being of present and future generations^8^. Kirchherr et al.^9^ collected more than 100 definitions of the circular economy concept and concluded that “the circular economy is a system that replaces the concept of ‘end-of-life’ with reduction, alternative reuse, recycling, and recovery of materials in production, distribution, and consumption, which is enabled by new business models and responsible consumers”. The central idea of agriculture is to promote the recycling of agricultural resources^5^. Notably, in the case of circular agriculture, it is more important to use such resources effectively than simply to recycle them.

To achieve the efficient use of agricultural resources and ensure sustainable agricultural development, the European Union (EU) is pushing for a zero-waste economy by 2030, focusing on reusing agricultural waste and achieving sustainable development. The European Commission promoted an initiative entitled the Green Deal in December 2019 to accelerate the transition to a circular economy for a cleaner and more competitive Europe^10^. These policy efforts have brought increasing attention to consumers’ roles in the transition toward more sustainable food systems. Many studies have examined consumers’ preferences for agricultural products and the factors influencing purchase decisions. For example, Villanueva et al.^11^ conducted a study on Iberian ham and found that consumer preferences were related to the feeding system used and objective quality clues. The study conducted by Salazar-Ordóñez et al.^12^ showed that consumers’ attitudes toward different types of olive oil play a key role in explaining their consumption.

Building on the growing interest in the role of consumers in sustainable agriculture, consumers’ purchasing and consumption behaviors with regard to sustainable food products have attracted a great deal of attention from academia and industry in recent years. In this context, it is necessary to briefly review the key theories in the field of consumer behavior and willingness to pay (WTP) for sustainable products. The Theory of Planned Behavior^13^ is a commonly-used theory in this field. It holds that individual purchasing behaviors are jointly influenced by three key factors: attitudes, which represent the individual’s overall evaluation of the behavior; subjective norms, which refer to the perceived social pressure to engage in or not engage in the behavior, and perceived behavioral control, which reflects the individual’s perception of the difficulty and ease of performing the behavior^13^. Additionally, the Value-Belief-Norm theory, first proposed by Stern et al.^14^, has been used to analyze consumers’ food-related behavior. In this theory, personal values influence beliefs, and beliefs in turn influence norms, which directly motivate an individual’s pro-environmental behavior^14,15^. In the context of sustainable food-related behavior, consumers with strong environmental values may believe that choosing sustainable food products helps to reduce environmental pollution, and this belief may lead them to form norms that prioritize purchasing such products.

Moreover, in the field of circular agricultural products, academic research has gradually increased in recent years. Many studies have focused on the development strategies^1,16^ and technological tools related to circular agriculture^17^, or on farmers’ perceptions and attitudes from a producer’s perspective^18^. For example, Atinkut et al.^19^ explored farmers’ WTP for a circular agriculture model under the “polluter pays” principle in central China and found that education, infrastructure, trust in family/neighbors, and environmental attitudes had a significant effect on WTP. Egan et al.^20^ investigated 1225 farmers in seven Northwest European countries about their perceptions and preferences regarding the properties and parameters for recycling-derived fertilizers. They found that the most desirable properties were common across the participating countries, including known nutrition, high organic content, product cost, and ease of use of recycling-derived fertilizers. In addition, income, sustainable recycling behavior, environmental perceptions, perceived usefulness-simplicity, and trust in government positively influenced farmers’ WTP. Danso et al.^21^ applied a choice experiment to assess African farmers’ WTP for manure sludge and municipal solid waste-based compost products, concluding that product price and quality, gender, experience, and household size could influence the WTP for recycled compost. However, few researchers have analyzed WTP and demand from the consumers’ perspective with regard to food produced by circular agricultural methods. Suárez-Cáceres et al.^22^ examined consumers’ WTP for aquaponic products in Spain and Latin America and realized that consumers’ income, environmental concerns, and knowledge of aquaponics affect their WTP for aquaponic products; a total of 60.5% of participants were willing to pay a higher price for such products. Villanueva et al.^23^ analyzed consumers’ preferences for food products produced in line with circular bioeconomy (CB) principles, including CB-related attributes, namely zero waste generation, carbon neutrality, sustainable water and soil management, biodiversity conservation, and contribution to the rural economy. This differs from the focus of our study, which aims to explore consumer demand for circular food products, their WTP, and consumer heterogeneity in order to provide some suggestions for relevant stakeholders in the EU for the promotion of circular products. On the other hand, evidence on consumers’ WTP for other circular agricultural products is sparse. Additionally, Jurgilevich et al.^24^ explored potential strategies for transitioning to a circular economy from the perspective of the food system, i.e., food production, food consumption, and food waste and surplus management. Nonetheless, there is still a lack of in-depth research on consumer demand and WTP for circular agricultural products. In addition, Zhang et al.^8^ mapped the relatively new but growing field of circular economy by systematically reviewing the literature, and the results indicated that food waste management is a prominent topic related to circular economy.

However, these existing studies lack analysis of consumers’ WTP and demand behavior with regard to circular agriculture and detailed research on factors affecting consumer behavior. Moreover, the role of consumers is essential in the transition to a circular economy in terms of making sustainable choices^24^. Furthermore, it is vital for governments to introduce related policies and for producers and marketers to understand consumers’ WTP for food products produced from a circular system as a means of promoting sustainable agriculture. Specifically, understanding consumers’ demand and WTP for circular agricultural products would allow EU policymakers to develop targeted policies such as subsidies or incentives for circular agriculture, and adjustments in product pricing mechanisms to better meet consumers’ expectations. This could promote the adoption of circular farming practices and contribute to the goals of the Green Deal, such as reducing greenhouse gas emissions and improving resource efficiency in the agricultural sector.

To fill these gaps in the research, consumers’ WTP and demand at a European level were measured by using an open-ended choice experiment (OECE), an approach which has been proven to be both novel and useful. A large sample of consumers from six EU countries was collected, and three food categories in the form of three case studies, i.e., pork from pig production, milk from cattle production, and bread from cereal production, were considered.

Hence, this study employs OECEs to examine consumers’ WTP and demand in six EU countries with regard to three types of food products produced by circular agriculture, with the specific aims of (1) examining the market potential for circular-labeled food products in Europe; (2) identifying cultural or socio-economic differences among the analyzed countries; (3) exploring cross-country variations in consumer preferences for circular food products across the three case studies; and (4) providing valuable insights for EU policymakers to promote circular agriculture.

## Results

Details of results with regard to the socio-demographic characteristics of the samples and frequency of food purchases are presented in the Supplementary Information (Supplementary Results section and Supplementary Tables 2 and 3). As for environmental attitudes, the details of the Principal Component Analysis (PCA) results and the environmental attitudes results are displayed in the Supplementary Information.

To summarize, the proportion of men and women was acceptable in accordance with the population distribution of each country, but Poland had a slightly higher proportion of women (59.52%). The age distribution was similar in most countries, but Croatia had a smaller proportion of people aged 35–44. The majority of respondents in all countries had completed secondary and university education and were full-time employees. Consumers in all countries stated that their financial situation was normal, and the majority of respondents’ income could cover their expenditure. The PCA results show that respondents in Italy, Hungary, and Croatia were more concentrated in the fourth quadrant, highlighting their ecocentric attitudes. However, respondents in Spain, Poland, and Belgium were more dispersed, with slightly more in the second quadrant, indicating that they hold anthropocentric attitudes.

### Aggregated and average demand for circular food products

**Fig. 1:**
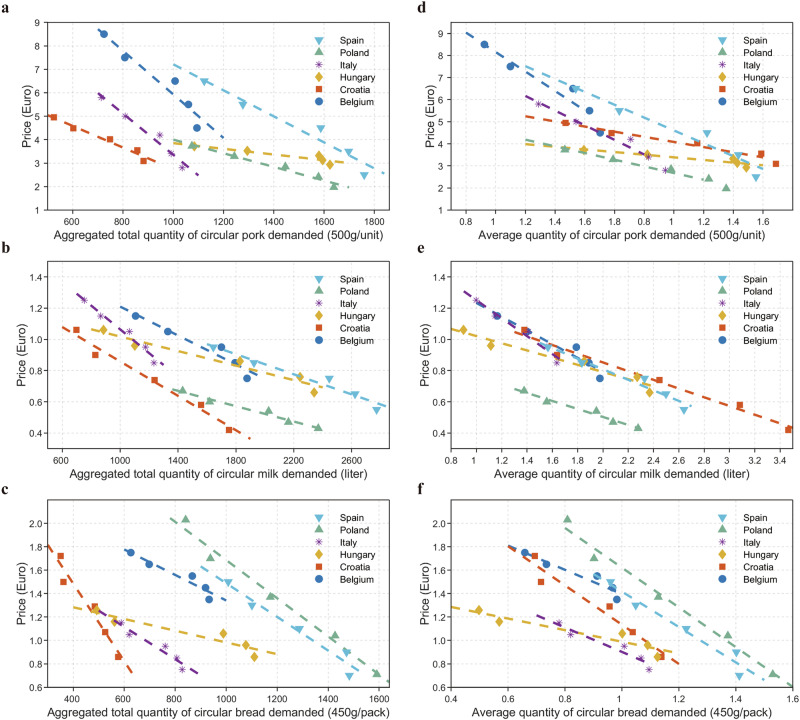
Observed aggregate and average demand for circular food products in six countries. **a**–**c** Aggregated total quantity of circular food products. **d**–**f** Average quantity of circular food products. It was calculated as the total quantity divided by the sample size of each country.

We summed individual demand at each price level for the three products from three farming systems and aggregated total quantities (Supplementary Tables 5–7). The demand curves for circular products are plotted based on these results and are displayed in Fig. 1. The results showed that the demand for the three circular products, both total and average, decreased with the increase in price in all six EU countries. This was consistent with the “law of demand”. This trend remained consistent across all demographic groups (gender, age, and education level) and environmental attitude groups (ecocentric and anthropocentric attitudes), as illustrated in Supplementary Tables 8–10 and Supplementary Figs. 4–7, which display the demand curves.

In terms of gender, we found that male demand curves were flatter than female demand curves, suggesting that men were more responsive to price changes, while in Poland, females were more price-sensitive. As for age, consumers aged 35–54 were more price-sensitive in Hungary, while young people in Spain (18–34 years of age) and older people in Poland (55 years or older) were price insensitive since their demand curves were steeper. Additionally, in Croatia, Poland and Italy, consumers with below university education levels were more price sensitive. Regarding environmental attitudes, in Hungary, Croatia, Poland and Spain, ecocentrists were more responsive to price changes.

### WTP for circular food products

**Fig. 2:**
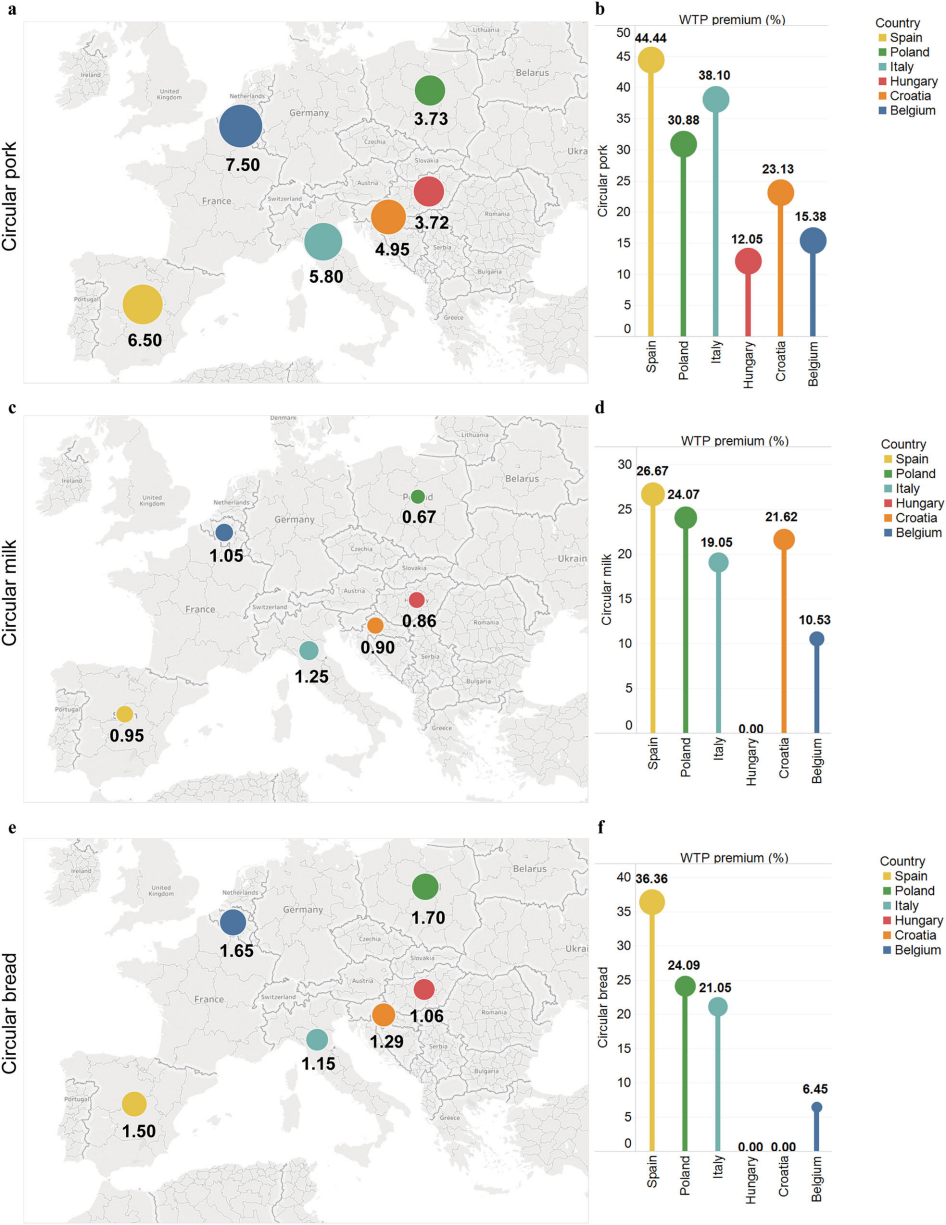
Consumers’ median maximum WTP (in monetary and percentage terms) for circular food products in six EU countries. **a**, **c**, **e** Participants’ median maximum WTP (€). They are based on the results in Supplementary Tables 11–13. As described in the “Methods” section, it is estimated as the maximum price at which respondents indicate a positive product quantity. **b**, **d**, **f** WTP premium in percentage terms (over conventional products of each country). They are based on Formula 1 mentioned in the “Methods” section.

Figure 2 shows consumers’ median maximum WTP (in monetary and percentage terms) for circular food products in six EU countries. Supplementary Tables 11–13 display the summary statistics of the quantity demanded for circular sliced pork, milk, and sliced bread in six EU countries. These are the basis for Fig. 2a, c, and e. Figure 2a suggests that the median maximum price that participants were willing to pay for a unit of circular pork (500 g) was €6.50 in Spain, €3.73 in Poland, €5.80 in Italy, €3.72 in Hungary, €4.95 in Croatia, and €7.50 in Belgium. In the case of circular milk (Fig. 2c), the median maximum price that consumers were willing to pay for a liter of milk was €0.95 in Spain, €0.67 in Poland, €1.25 in Italy, €0.86 in Hungary, €0.90 in Croatia, and €1.05 in Belgium. As for circular bread (Fig. 2e), the median maximum WTP of consumers for a pack of circular bread (450 g) was €1.50 in Spain, €1.70 in Poland, €1.15 in Italy, €1.06 in Hungary, €1.29 in Croatia, and €1.65 in Belgium.

In terms of the maximum WTP premium (median) in percentage terms compared to the price of a conventional pork loin of the same size (Fig. 2b), consumers in Spain had the highest WTP premium (in percentage terms) for 500 g of circular pork loin at 44.44%, followed by Italy at 38.10%, Poland at 30.88%, Croatia at 23.13%, and Belgium at 15.38%, while consumers in Hungary showed the lowest premium at 12.05%. The maximum WTP of respondents in Spain for 1 liter of circular milk was the highest at 26.67%, followed by Poland at 24.07%, Croatia at 21.62%, Italy at 19.05%, and Belgium at 10.53%, while Hungarian consumers were willing to pay a premium percentage of 0%, i.e., the WTP for circular milk was equal to the price of conventional milk (Fig. 2d). In addition, the maximum WTP premium of participants in Spain for circular bread (450 g) was the highest at 36.36%, followed by Poland at 24.09%, Italy at 21.05%, and Belgium at 6.45%. Participants in Hungary and Croatia had the lowest premiums at 0% (Fig. 2f). Regarding these three products, Spanish consumers displayed the highest WTP premiums (in percentage terms), whereas Hungarian consumers exhibited the lowest premiums. In addition, the WTP premiums of consumers in Spain and Italy were higher than those in Belgium.

### Factors affecting the demand for the circular food products

**Fig. 3:**
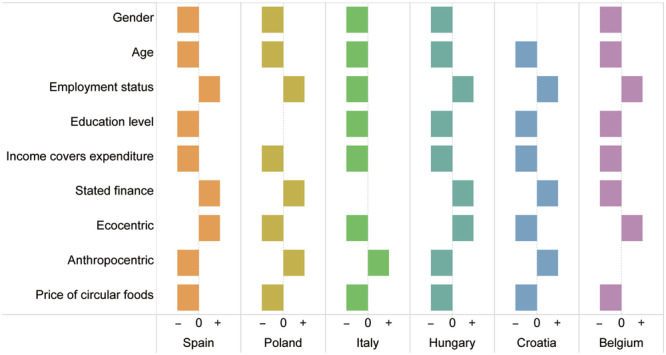
Summary of Negative Binomial (NB) results of three circular food products in all countries. The y-axis represents the significant factors influencing consumer demand, and the x-axis represents the quantity of circular food products that consumers in each country are willing to buy. The “+” sign implies a positive relationship between the factors and quantities, while the “−” sign denotes a negative relationship.

Supplementary Tables 14–19 present the results of the Negative Binomial (NB) regression for each country, mainly showing the statistically significant results for each group of variables due to word limitations, and Fig. 3 summarizes the results for the three circular food products in all countries. Our results revealed that in all six countries, age, employment status, income availability to cover monthly expenditure, environmental attitudes, and price of circular food products were the key factors influencing quantity demanded of the circular products considered in this study. Specifically, in all countries, younger consumers expressed a willingness to purchase more circular products than older people. Employment status was associated with the quantity of circular products. As expected, respondents whose monthly income rarely and never covered their household expenditure expressed a willingness to purchase a lower number of circular products when compared to those whose income always covered expenditure. Consistent with expectations, the price of circular products was negatively associated with the number of circular products purchased. Therefore, the hypotheses H2b, H2c, H2e, and H3 are supported.

In addition, ecocentric consumers expressed a willingness to purchase a greater number of circular products in Spain, Hungary, and Belgium. Therefore, the hypothesis H1 is supported. Nevertheless, in Poland, Italy, and Croatia, ecocentric consumers expressed a willingness to purchase a lower number. Results from Supplementary Table 20 reveal that ecocentric consumers had a positive correlation with vegetarian, vegan, and flexitarian diets, and a negative correlation with non-restricted diets in all countries. Ecocentric consumers perceived vegetarianism, veganism, and flexitarianism to be more environmentally sustainable, while those on non-restricted diets were perceived as less sustainable. Supplementary Table 20 also shows that ecocentric attitudes were positively correlated with organic and circular farming, and negatively correlated with conventional farming in five countries, while in Italy it was found that there was a negative correlation between ecocentric attitudes and circular farming.

Somewhat surprisingly, our results revealed that males tended to report a willingness to purchase more circular products than females in five countries (with the exception of Croatia). As expected, people with a high level of education expressed a willingness to purchase more circular products than those with a low level of education (with the exception of Poland). Consequently, the hypothesis H2d is supported, whereas the hypothesis H2a is not supported. The stated financial situation was found to influence the quantities of purchases (with the exception of Italy). Participants who stated that they were in a good financial situation expressed a willingness to purchase larger quantities of circular products than those who stated that they were in a difficult financial situation in Spain, Poland, Hungary, and Croatia. However, consumers who stated that they were in a good financial situation expressed a willingness to purchase smaller quantities in Belgium.

## Discussion

In general, circular agriculture production has higher costs than conventional agriculture; therefore, it is interesting and informative to compare such costs with the premium of WTP for circular food to see if this production method pays off. We found that for circular pork loin, consumers in EU countries were willing to pay a premium ranging from 12.05% to 44.44%, with an average premium of 27.33%. This wide range highlights significant variations between countries, emphasizing the importance of interpreting averages with caution; for circular milk, the WTP premium ranged from 0% to 26.67%, with an average of 16.99%; for circular bread, consumers were willing to pay a premium ranging from 0% to 36.36%, with an average premium of 14.66%. Consequently, it would be valuable to further determine the costs associated with these three types of circular production in terms of how much higher it would be than the cost of conventional agricultural production. In addition, the largest portion of consumer prices is determined by retail and gross margins (and those of other intermediaries such as slaughterhouses). The margin of the farmer in this is extremely slim. In fact, if the premium profit for added-value products (e.g., organic or circular) could be fully passed on to the farmer, then price increases for such products could be kept to a minimum and their effects on farmers (and the environment) would be maximized. That is, it would enable the farmer to obtain the majority of the profit from the premium portion of the price in the context of ensuring the cost of circular production.

There is market potential for circular farming food in Europe, as consumers expressed a willingness to purchase and pay a premium for food products with circular labels. Among the three products studied, Spanish consumers showed the highest WTP premiums (in percentage terms), whereas Hungarian consumers exhibited the lowest premiums. This disparity might be linked to differences in economic development. Spain has a higher Gross Domestic Product (GDP), a higher GDP per capita^25^, and relatively high disposable income levels, which may lead consumers to prioritize sustainability over cost. In contrast, Croatia and Hungary, with relatively lower GDP, may face economic constraints that limit consumers’ interest in circular products. Additionally, cultural and behavioral factors may play a role. For example, the WTP premiums in Spain and Italy were higher than those in Belgium. This could be attributed to the greater likelihood of Spanish and Italian consumers considering the environmental impact of their food choices compared to Belgian consumers^26^. Another contributing factor may be the relatively higher average cost of food items in Belgium compared to Spain. Since pork loin, milk, and bread are already more expensive in Belgium than in other countries, consumers may be less willing to pay an additional premium for circular products.

Next, the results indicated that younger consumers expressed a willingness to purchase more circular food products than older people. This supported the findings of numerous studies indicating that young consumers tend to purchase more sustainable food products^27–29^. Younger consumers are more sensitive and concerned about issues related to sustainable development^30,31^ than older consumers, and they are increasingly interested in more sustainable behavior such as buying green products^32^. In addition, the younger generation has acquired relevant environment-related knowledge through environmental education and is inclined to adopt practices that respect the natural environment^33^. As expected, respondents whose monthly income rarely or never covered their household expenses expressed a willingness to purchase fewer circular products compared to those whose income consistently covered their expenses. Likewise, previous research showed that disposable income plays a major role in the quantity of organic products purchased^34^. The stated financial situation was found to influence the quantities of potential purchases (with the exception of Italy). Participants in Spain, Poland, Hungary, and Croatia who stated that they were in a good financial situation expressed a willingness to purchase larger quantities of circular products than those who stated that they were in a difficult financial situation. Similarly, this finding tied in with the idea that consumers with a higher income might purchase more sustainable food products^35^. Nevertheless, the results also revealed that consumers in Belgium who stated that they were in a good financial situation expressed a willingness to purchase smaller quantities, which was not in line with our expectations. One possible explanation for this could be that some consumers in Belgium did not accurately assess and express their financial situation. Additionally, in our case study, the stated financial situation was not found to affect Italian consumers’ purchases of circular food, which may stem from cultural or interpretative biases. Italian respondents may exhibit optimism or conservatism when self-assessing their financial status, reducing the reliability of this indicator in explaining purchasing behavior. Additionally, a report by Torjusen et al.^36^ suggests that Italian consumers of organic food (a sustainable product) tend to prioritize ethical and idealistic values over financial considerations. A similar pattern may apply to circular food products, where ethical motivations outweigh financial concerns. However, these hypotheses require further investigation. As expected, the price of circular products was negatively associated with the quantity of circular products purchased, demonstrating that the higher the price, the lower the demand, which is in line with demand theory. This also supported the findings of many other scholars^37–39^.

We found that ecocentric consumers tended to express a willingness to purchase more circular products in Spain, Hungary, and Belgium. One possible explanation is that individuals with an ecocentric attitude are more likely to wish to protect the environment in line with their values compared with those with an anthropocentric attitude^40^. Nevertheless, our findings revealed that ecocentric consumers in Poland, Italy, and Croatia expressed a relatively low willingness to purchase circular food products. This result may be due to several factors. One possible explanation is the well-documented attitude-behavior gap, where individuals with strong pro-environmental attitudes do not always translate these attitudes into actual purchasing behavior for sustainable products^38^. While ecocentric consumers may hold favorable views with regard to sustainability, this does not necessarily result in higher demand for circular food products. Another potential reason is that some ecocentric consumers may perceive sustainability as achievable through means other than food innovation. For instance, they may prioritize other sustainable practices such as reducing food waste or supporting local food systems over purchasing circular products. This perspective may stem from their deeper understanding of environmental sustainability, as indicated by Supplementary Table 20, which highlights differences in sustainability perceptions among respondents. To address this issue, policymakers and authorities in Poland, Italy, and Croatia could focus on emphasizing the ecological benefits of circular products to ecocentric consumers. Increasing awareness through education and training could help to bridge the knowledge gap and encourage greater adoption of circular products. This approach is supported by previous research which has shown a positive correlation between sustainability education and pro-environmental behavior^41^. By enhancing consumer knowledge, it may be possible to align their attitudes with their purchasing behavior and increase demand for circular food products.

Somewhat surprisingly, our results revealed that males expressed a willingness to purchase more circular products than females in most countries (with the exception of Croatia), contrary to many studies’ conclusions which indicated that women were more likely to buy eco-products^42,43^. However, our result was similar to those of Castro and Chambers^44^, who estimated consumers’ willingness to eat an insect-based product in thirteen countries and found that in most countries, men were more willing to try new sustainable products than women. Similarly, Verbeke^45^ concluded that men were twice as likely as women to adopt insects—a novel and sustainable food—as a meat substitute. While these studies focus on insect consumption, which is influenced by unique factors such as neophobia, their findings suggest that men may be more open to trying new eco-friendly food products. This could reflect broader trends in sustainable behavior; however, caution is warranted when generalizing these results to other sustainable practices such as circular product consumption, due to differences in context and consumer perceptions. This observation may also be linked to evolving societal roles, as men increasingly take on food purchasing responsibilities in modern households^46^. Additionally, several studies have pointed out that men were more knowledgeable and concerned about environmental issues and had a more positive attitude toward sustainable purchasing than women^47^. Nevertheless, it is important to recognize that gender-related differences in sustainable behavior can vary significantly depending on cultural, social, and economic contexts. Further research is needed to explore these dynamics in the context of circular product consumption and to better understand the underlying factors driving these trends.

In addition, our study expands the theoretical understanding of consumer behavior, particularly in the context of sustainable food-related behavior. Traditional consumer behavior theory often displays limited and predefined options when studying consumers’ sustainable choices, which restricts the scope of consumer decision-making. By using OECEs, this study allows consumers to express their purchase quantities independently, liberating them from the constraints of conventional methods. Specifically, when examining EU consumers’ purchasing behavior with regard to circular food products, participants were not restricted to a limited set of product combinations. Instead, they could freely decide the quantity of products such as circular sliced pork loin, milk, and bread, based on their consumption needs and their understanding of circular food practices. This approach provides a new perspective for developing a more realistic and explanatory framework for consumer decision theory.

Furthermore, our case study using OECEs revealed significant heterogeneity among consumer groups in their responses to circular agricultural products. For example, young consumers, men, and high-income groups, demonstrated a stronger inclination to purchase circular food products, reflecting a heightened willingness to support sustainable consumption. These findings complement existing studies and highlight the importance of considering diversity within consumer groups. The insights gained from OECEs inspire future theoretical research to focus not only on aggregate consumer behavior but also on the nuanced differences among subgroups. This shift in focus can lead to a more comprehensive understanding of sustainable consumption patterns and inform the development of targeted interventions.

The findings of this study also provided valuable insights for EU policymakers and stakeholders aiming to promote circular farming. One important recommendation is the revision of the Nitrates Directive (91/676/EEC) to reflect three decades of technological advancement. The current inflexible and outdated requirements should be updated to allow the refinement of manure into organic fertilizer products as an alternative to chemical fertilizers. This change would facilitate the transition from “waste” to “resource”, advancing the goals of circular agriculture. Additionally, EU policymakers should establish clear definitions for circular food products, similar to those for organic farming. Clear guidelines would help agricultural producers adhere to circular practices and enable consumers to identify and choose circular products, thereby promoting sustainable purchasing and consumption behavior.

Another critical area for action is addressing the higher production costs associated with circular agriculture compared to conventional farming. Policymakers should calculate these costs and implement differentiated subsidies to ensure profitability for producers. This approach would encourage more farmers to adopt circular practices and support the growth of the circular economy.

In terms of market segmentation and marketing strategies, this study identified five key consumer segments with a higher willingness to purchase circular food products: young consumers, men, higher-income consumers, consumers with higher education levels, and business owners and employees. Policymakers and businesses should tailor marketing strategies to these groups. For example, enterprises could develop circular food products that appeal to young consumers and promote them through social media and other digital platforms. Supermarkets and other marketplaces could prominently display circular food products and use targeted campaigns to raise awareness with regard to these segments.

Finally, consumer education and awareness-raising are essential, particularly in countries such as Poland, Italy, and Croatia, where ecocentric consumers show lower willingness to purchase circular food. EU policymakers should launch educational initiatives to ensure that consumers are well-informed about the benefits of circular agriculture. Public service advertisements on television, radio, and other media could further popularize circular agriculture and enhance public acceptance. Additionally, supermarkets and other marketplaces should be encouraged to increase the visibility of circular food products through display boards, brochures, and promotional posters. These efforts can help to bridge the gap between consumer awareness and action, ultimately driving the adoption of circular food products across Europe.

Consumers play an essential role in sustainable agricultural systems, and understanding their WTP and preferences for food products from circular agriculture is crucial in terms of promoting the overall sustainability of agricultural systems. In conclusion, the findings reveal a market niche for these products, with consumers willing to pay a premium over conventional products. Spanish consumers exhibited the highest WTP premiums, while Hungarian consumers showed the lowest premiums. Factors such as socio-demographic characteristics, environmental attitudes, and price levels influenced the quantity of circular food products that consumers were willing to purchase.

This study has several limitations. Firstly, it focused solely on six EU countries, excluding other regions. Future research could explore additional regions, such as North America, South America, Oceania, and Asia, to determine if similar results can be obtained. Secondly, the online panel survey method, while providing a broad and diverse sample, may have introduced biases due to differences in Internet access and technological proficiency, such as over-representation of tech-savvy individuals or urban populations. Future studies could address these limitations by employing alternative or complementary sampling methods, such as face-to-face surveys in rural areas or hybrid approaches, to ensure a more balanced representation of the population. Additionally, the OECE method relies on self-reported data, which is susceptible to social desirability bias. Respondents may overstate their willingness to purchase circular products to align with perceived societal expectations. The hypothetical nature of the OECE method may also lead to hypothetical bias, potentially resulting in an overestimation of demand. Future research could adopt a mixed-method approach, combining OECE with observations of actual market behavior, to provide more accurate and reliable insights. Despite these limitations, the OECE method, which included circular, organic, and conventional food products, closely simulated real market conditions and provided valuable insights into consumer behavior. Overall, this study highlights the market potential of food produced through circular farming practices in Europe and provides valuable insights for policymakers and stakeholders in terms of promoting circular farming systems to achieve sustainable agriculture.

## Methods

### Selection of study area

Our study spans six countries—Spain, Italy, Belgium, Poland, Croatia, and Hungary—across four distinct geographical regions. Spain and Italy are located in Southern Europe and reflect the economic and agricultural landscapes of the region. Belgium is located in Western Europe with a developed economy. Poland, located in Central Europe, stands as one of the largest agricultural producers within the EU. Croatia and Hungary, situated in Central and Eastern Europe, exhibit developing agricultural sectors.

Due to different stages of economic development and cultural heritage, consumers in these countries may have different levels of awareness and acceptance of circular agricultural food. Given that our study covers Western Europe, Southern Europe, and Central and Eastern Europe, encompassing different economic development levels and cultural backgrounds, this diversity provides a comprehensive perspective.

The selection of these countries enables a better understanding of the market potential of circular agricultural products in Europe and the exploration of consumers’ behavior in different socio-economic contexts, providing valuable cross-national comparisons for policymakers. Additionally, each of these countries has different policies and regulations related to agriculture, sustainability, and food production, allowing us to explore consumers’ demand and preferences for circular food products in various contexts.

### Selection of food products

Three food categories, i.e., pork from pig production, milk from cattle production, and bread from cereal production, were considered in the form of three case studies. One of the reasons these particular food products were selected was the relative importance of each of the sectors within the EU from a production point of view. The 142 million pigs reared across the EU are the largest livestock category followed by cattle (76 million), and the EU pork sector alone accounts for nearly half of the EU’s total meat production^48^. In addition, the EU is the world’s second-biggest producer of pork after China and the biggest exporter of pork and pork products^49^.The dairy sector is the second-largest agricultural sector in the EU, accounting for over 12% of total agricultural output^50^. The harvested production of cereals (including rice) across the EU was 299.4 million tons in 2019, with 119.1 million tons of common wheat and spelt, equivalent to 44% of all cereal harvests^51^. Another reason for selecting these three products was that they are well-known and frequently purchased by participants, which reduces the novelty bias when making choices in the contingency market^52^. In addition, these sectors have high levels of emissions. Thus, it is interesting and valuable to focus on them.

### Definition of food produced by circular agriculture in our case studies

In agriculture, carbon, nitrogen, phosphorus, and potassium are essential for maintaining fertile and healthy soils and for allowing plants to grow and develop fully. However, in the current system, soils are being depleted of carbon and losing valuable nutrients, leading to river and air pollution and contributing to greenhouse gas emissions. This is because of the inefficient use of nutrients in our agricultural systems. More than half of the nitrogen and phosphorus used comes from non-renewable sources, yet only one out of every five tons of nitrogen entering the EU agri-food chain is actually converted into food for human consumption; the situation is similar in the case of phosphorus and potassium. In addition, poor soil management practices have led to carbon loss from the soil, worsening the environmental situation. Our research aimed to close carbon-nitrogen-phosphorus loops by reconnecting nutrient and carbon flows between conventional agro-pillars (three pillars: agro-processing, animal husbandry, and plant processing) through agro-processing. In addition, understanding consumers’ WTP and demand is essential when it comes to incorporating these innovations.

Circular production in the pig industry studied in our research refers to the treatment of pig slurry and manure to produce bioenergy (biogas) and bio-based fertilizers using a combination of technologies^53^. In the case of circular cattle farming, dairy farms use wastewater to produce algae as a new source of protein (animal feed), and residues from the food industry (dairies and dairy plants) are used to produce fertilizer and build soil fertility^54^. The microalgae biomass used for animal feed not only improves food security for humans and animals, but also reduces greenhouse gases produced by agricultural activities^55^. In the case of circular bread products, grains for flour are grown using an integrated crop management system that uses low-carbon techniques such as no-till, crop rotation, and cover crops to increase soil fertility and organic matter content, and fertilizes the crop with recycled bio-based fertilizer^17,56^.

### Design of the open-ended choice experiment

The WTP estimates represent the price premium or the maximum amount that a current or potential consumer is willing to pay for a product or a good^57^. As an approach to measure WTP, OECE combines the advantages of experimental auctions (EAs) and discrete choice experiments (DCEs). Compared to DCEs, OECEs not only allow respondents to choose the products they like, but also indicate the quantities they are willing to purchase at different price levels. This approach therefore better simulates the real market conditions and provides richer information by capturing both preferences and demand information. Unlike EAs, whose complexity and reliance on the physical environment limit their application in large-scale cross-national studies, OECEs can use online questionnaires, which have both flexibility and low implementation costs that make them particularly suitable for cross-national research.

Respondents are presented with multiple products sold at different prices (as in DCEs) and asked to indicate the number of products they would be willing to purchase at different price combinations. Thus, their responses are open-ended (as in EAs)^58,59^, which could simulate a realistic scenario of consumer purchases. Therefore, OECEs have been employed by some scholars in food-related research^59–61^. The major difference between OECEs and DCEs is that in the latter, participants are asked to choose only the product they prefer, whereas in OECEs, participants select both the product and its quantity^60^. Since respondents can purchase any non-negative quantity at different price levels, researchers are able to estimate not only the respondents’ WTP for a unit of the product, but also the participants’ entire demand curve^58,59^.

**Fig. 4:**
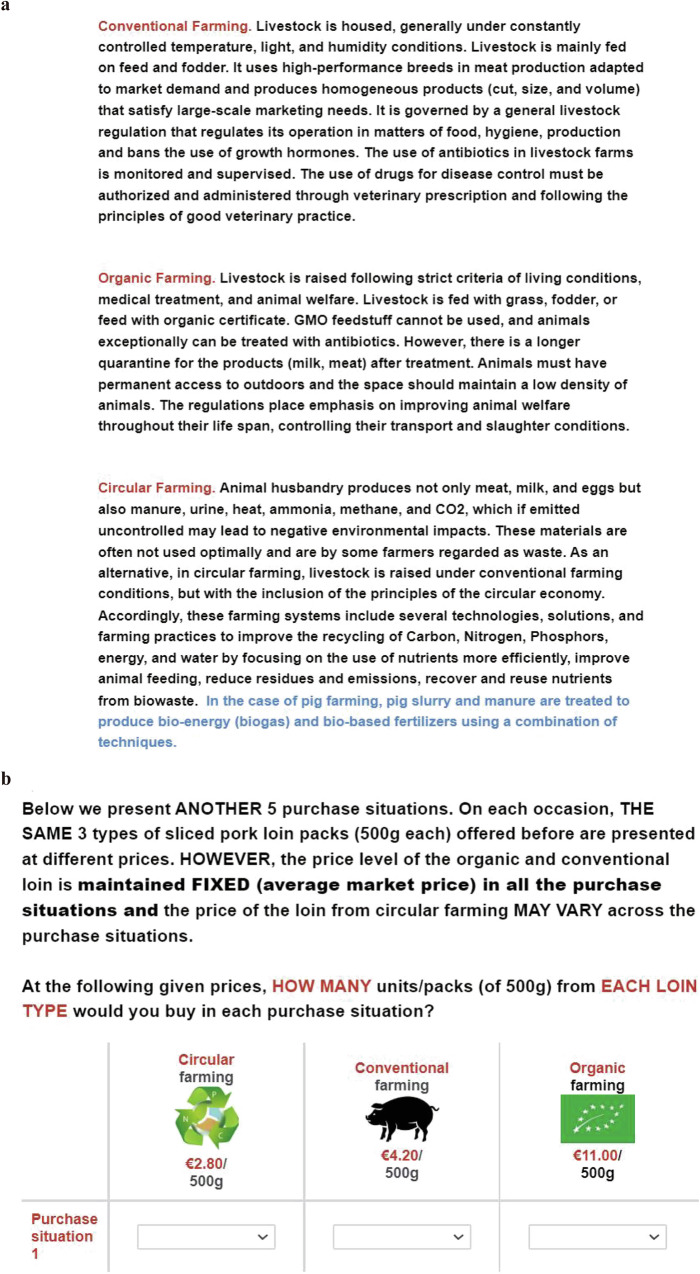
An example of sliced pork loin in the OECE, and definitions of various farming systems for pork. **a** Definitions of various farming systems for pork that respondents were presented with before making choices in OECEs. **b** An example of sliced pork loin in the OECE.

Three OECEs for pork loin, milk, and bread were designed. In addition to circular food products, organic and conventional products (substitutes and competitive agricultural systems) were also introduced, since previous studies have suggested that novel products should be evaluated in the context of substitute and competitive commodities that consumers can purchase in the market^59^. This means that such an approach could simulate the real market to reduce hypothetical bias. In the case of circular agriculture, we introduced three innovations. Evidence of order effects in DCEs exists^62^, and similar effects may also occur in OECEs. Therefore, respondents in our study were presented with three products (pork, milk, and bread) in OECEs in a randomized order. The order of the products within each purchase situation and the purchase situations presented to consumers were also randomized to mitigate potential order effects. There were five purchase situations in each case, and in each purchase situation, these products were presented at different prices. However, consistent with the research of Wongprawmas et al.^59^ and Corrigan et al.^58^, in all purchase situations, the price level of organic and conventional products was fixed (average local market price), while the price of products from circular farming varied across purchase situations. The price vector for each product category and farming system was determined based on the specific products (type and weight): 500 g sliced pork loin, 450 g sliced bread, and 1 liter of milk. Price levels and product size were identified after a deep review and comparison with similar products at the market level in each country. The price vectors were presented in Supplementary Table 1. For example, in Spain, participants were offered an array of potentially binding prices for 500 g packages of pork loin (ranging from €2.50 to €6.50 in €1.00 increments), and 500 g packages of conventional and organic pork loin would always be available at the €4.50 and €10.50 field prices, respectively. All respondents were presented with this information and the definitions of different farming systems (see Fig. 4) and were asked to indicate how many products they would purchase in each purchase situation. If they were not interested in these products, they could indicate a “zero” quantity (no purchase)^59^.

In the case of the circular farming systems, because the products analyzed were not available in the marketplace compared with conventional and organic products, the price levels were set close to the average price level of conventional products (which were the products to be compared with) by including some variation downwards and upwards^59^. As a result, the identified price vector for products from circular farming was considered to be relatively close to the average price of the conventional alternative in the real marketplace. These price ranges were selected to encompass the potential values of respondents’ WTP^59^. Given the variation in the average market prices of substitutes across these EU countries, different price ranges for circular food were adopted for each country. Bread and milk are products frequently purchased by consumers, and many substitutes usually exist in the bread and milk market. As a result, consumer demand is likely to be more price elastic, indicating that they are more responsive to price changes. That is, the magnitude of the percentage difference in the price of products from different systems is important to consumers when purchasing a product on a frequent basis^63^. In this context, in our research, the price ranges for circular bread and circular milk were set narrower than those for circular pork loin. When collecting the data, the prices were in the local currency of each country, and the currencies of Poland (Polish złoty), Hungary (Forint), and Croatia (Kuna) were converted into Euros when analyzing the data. An example of sliced pork loin in the OECE is displayed in Fig. 4. Examples of milk and bread are presented in Supplementary Figs. 1 and 2.

### Questionnaire design and data collection

To measure consumers’ WTP and demand for circular pork loin, bread, and milk, a semi-structured questionnaire was developed. The questionnaire consisted of four sections: (1) open-ended choice experiments and frequency of food purchases; (2) consumers’ environmental attitudes; (3) opinions on the sustainability of different production systems and dietary patterns; and (4) socio-demographic information, including gender, age, employment status, education, and income status.

The study was conducted in six EU countries: Spain, Italy, Belgium, Poland, Croatia, and Hungary (see Supplementary Fig. 3). A pilot survey was conducted in each country with a sample of 50 consumers to identify potentially confusing points, enabling the researchers to modify the questionnaire and check for consistency before the formal distribution. Finally, a total of 5591 respondents participated in the survey in these six EU countries. Excluding invalid questionnaires (e.g., participants whose response time was too short, less than 3 minutes), the remaining 5289 valid questionnaires were used for analysis.

The data were collected from June 2021 to January 2022 using the Qualtrics market research consumer panel in five countries (Spain, Italy, Belgium, Poland, and Croatia) and the Netpanel market company in Hungary. These providers ensured a representative sample in each country through a quota-sampling approach. Quotas were based on key demographic variables such as age and gender to reflect the national demographics of each country. The questionnaire was initially presented in the local language to ensure clarity and accuracy for all participants and was subsequently translated into English.

The participants participated in this survey voluntarily and anonymously. At the beginning of the questionnaire, participants received written informed consent that outlined the survey’s main content and purpose, their right to withdraw at any time, and their privacy protection (see the full questionnaire in the Supplementary Information). The questionnaire was authorized by CREDA’s Ethics Committee and was conducted in line with the ethical norms of social science research. The selection criteria were participants who were at least 18 years old and food purchasing decision-makers in their households. To enhance transparency, Table 1 presents the technical details of the survey.

**Table 1:** Technical specifications of the survey

Specification	This Survey
Study area	Six EU countries: Spain, Italy, Belgium, Poland, Croatia, and Hungary
Survey method	Online semi-structured questionnaire
Survey content	(1) OECEs and frequency of food purchases; (2) environmental attitudes; (3) opinions on the sustainability of different production systems and dietary patterns; and (4) socio-demographic information
Sample size	5591 respondents; 5289 valid questionnaires
Sampling method	Stratified sampling
Weighting	The total number of consumers surveyed was stratified by gender and age according to the demographic characteristics of each of the six countries
Exclusion criteria	Participants whose response time was too short, less than 3 minutes
Selection criteria	Participants who were at least 18 years old and food purchasing decision-makers in their households
Period of survey	June 2021 to January 2022

### Measuring consumers’ WTP and factors affecting demand for food products from circular farming

Attitude can be regarded as a psychological tendency expressed through a certain degree of favor or disfavor with regard to a particular entity^64^. Environmental attitudes are defined as “evaluative beliefs, affect, and/or behavioral intentions about environmentally related activities or issues”^65^. Many studies have identified environmental attitudes as a crucial antecedent that influences environmental behavior^66,67^. For example, Varah et al.^68^ found that people who display ecocentrism are more likely to exhibit behaviors that promote environmental protection. Therefore, in this research, consumers’ environmental attitudes were measured to explore their sustainable behavior and food choices. We propose the following hypothesis:

H1: Environmental attitude positively and significantly influences consumers’ purchase of circular food.

The New Ecological Paradigm (NEP) scale is becoming increasingly popular and has been widely used to measure consumers’ environmental attitudes. In our study, a reduced version of the original NEP scale was employed to measure consumers’ attitudes with regard to the environment, including only the statements relating to ecocentric and anthropocentric attitudes. It consisted of 8 statements expressing positive (4 statements) and negative (4 statements) attitudes and evaluations of the environment, which allowed us to analyze the relationships between subjects’ beliefs about humans and nature^69^. The respondents were asked about their environmental attitude via a 9-point Likert scale (from 1 = disagree very strongly to 9 = agree very strongly; 5 = neutral) *(Q: How much do you agree or disagree with the following statements?)*.

PCA was used to analyze environmental attitudes. The 8 specific items on the reduced NEP scale enabled the identification of latent dimensions related to consumers’ behavior toward the environment. Ecocentric consumers and anthropocentric consumers were identified in this way, which was consistent with Torres et al.^69^. The first component was identified as the ecocentric attitude, which was mainly determined by the statements from items 5–8, i.e., *“Item 5. Plants and animals have as much right to exist as humans”; “Item 6. The balance of nature is very delicate and easily alterable”; “Item 7. If things continue as they are, we will soon face a major ecological catastrophe”; and “Item 8. Despite our special abilities, humans are still dependent on the laws of nature”*. This first component (ecocentric) reflected a pro-environmental attitude. Ecocentric attitudes were nature-centered; they indicated a belief that non-human nature had intrinsic value^70^, that humans were one of the components of the whole natural system, and that humans should obey the laws of nature. They sought to strike a balance between human beings and natural ecosystems. The second component was related to the anthropocentric attitude, as reflected in the statements from items 1–4, i.e., *“Item 1. The balance of nature is strong enough to deal with the impact caused by economic development”; “Item 2. Over time, humans can learn how nature works to be able to control it”; “Item 3. Human ingenuity will ensure that we do not make the earth an uninhabitable place”; and “Item 4. Humans have the right to modify the environment to adapt it to their needs”*. Anthropocentric attitudes were human-centered; they indicated a belief that humans were the most important entity on earth^71^ and that humans could transform nature to suit their needs. The Kaiser-Meyer-Olkin (KMO) test and Bartlett’s test were performed before conducting the PCA.

Numerous studies have shown that consumers’ socio-demographic variables (e.g., gender, age, income, employment status, and education level) play a key role in their sustainable behavior with regard to food products. For example, Li & Kallas^72^ conducted a meta-analysis of 80 papers to measure consumers’ WTP for sustainable food products and found that females exhibited a higher WTP than males. Additionally, females and young consumers exhibited a higher intensity (the frequency or quantity of organic food consumed) in terms of organic food consumption^27,28^. However, some studies have suggested that older people display more sustainable consumption behavior than young adults. This may be because when young adults buy food, they prefer cheap and tasty takeaways without really considering the production line^73^. Some studies have found that monthly income positively affected the WTP for organic food^74^, while others have shown that income mostly influences the quantities of organic products purchased rather than consumers’ WTP^35^. Based on these findings, we propose the following hypotheses:

H2a: Females are more likely to purchase more circular food.

H2b: Young people show a higher willingness to purchase more circular food.

H2c: People with a higher income tend to purchase more circular food.

Another commonly studied factor is the level of education. Past studies have suggested that consumers with higher levels of education tend to buy larger quantities of organic food^75^. Moreover, education level and employment status (employed) positively influenced the likelihood of buying organic food^28^. Thus, the following hypotheses are proposed:

H2d: Consumers with higher levels of education are more likely to purchase more circular food.

H2e: Employment status positively influences the likelihood of buying circular food.

In addition, empirical studies have provided substantial evidence with regard to the influence of food-related factors on consumer purchase decisions. For instance, numerous papers have demonstrated that price is a crucial factor influencing consumers’ behavior with regard to sustainable food products^76,77^. Salazar-Ordóñez et al.^78^ found that price differences have a fundamental impact on consumer choices. In the case of olive oil, demand is almost inelastic when the price difference is within 1 euro per liter; however, once the price difference exceeds this range, demand for high-quality products falls sharply, indicating that it becomes highly price elastic. Therefore, the following hypothesis is put forward:

H3: Price has a significant negative impact on consumers’ purchase of circular food.

Therefore, in the questionnaire we included consumers’ environmental attitudes, socio-demographic variables (gender, age, income status, education level, and employment status), and the prices of circular, organic, and conventional food products in order to explore how they affect consumers’ sustainable behavior. Regarding income status, two questions were introduced. Firstly, respondents were asked to state their perceptions regarding their current financial situation using a 10-point Likert scale (from 1 = very difficult to 10 = very good). Secondly, they were asked to state how often their monthly income could cover their household expenditure (1 = always; 2 = very often; 3 = sometimes; 4 = rarely; and 5 = never).

In order to investigate consumers’ food purchasing frequency, participants were asked to answer questions about how often they shopped for groceries (Food in general; sliced pork loin; milk, and bread). Options included “daily”, “2–3 times a week”, “once a week”, “2–3 times per month”, “once a month or less”, and “never”.

### Data analysis

In this study, PCA was employed to analyze the environmental attitude variable. Descriptive analysis was also adopted. In addition, demand analysis and WTP analysis were conducted. Individual demand at each price level for sliced pork loin, milk, and sliced bread was summed, and the aggregated quantities were presented. The market demand curve for each product was the horizontal sum of the individual demand curves^79^. Due to the count nature of the quantity demanded, Poisson regression, Negative Binomial regression (NB), Zero-inflated Negative Binomial regression (ZINB), Zero Inflated Poisson (ZIP), or the Double-hurdle model could be employed^61^.

The Poisson regression model is used for counting data when the mean and variance are equal. It is not suitable for overdispersed data, where the variance is greater than the mean^80^. In comparison, NB regression is a suitable model for analyzing overdispersed data^81^. In our study, the variance and mean of the dependent variables (the quantity demanded) were different, and there was overdispersion; therefore, NB regression was more flexible and appropriate than Poisson regression. According to previous studies, the Vuong test can be employed to compare the ZINB model with the NB model to determine which was more suitable. When the *Z*-value obtained from the Vuong test is statistically significant, it suggests that the zero-inflated model should be used instead of the standard NB model when analyzing data^82^. In our case, the results of the Vuong test for our dependent variable indicated that the *Z*-value was not significant. Therefore, we opted to use the standard NB model for our analysis.

The traditional NB regression model was based on a Poisson-gamma mixture distribution^83^. STATA 17.0 software was used to conduct the NB regression. In the models, the dependent variables were the quantity demanded for three circular food products in each country. The independent variables were gender, age, education level, employment status, income covers expenditure, stated financial situation, environmental attitude (using the NEP scale), and price (prices of circular, conventional, and organic products).

We obtained the summary statistics for individual quantities of circular sliced pork, milk, and sliced bread demanded in six countries. The consumers’ maximum WTP was measured, and it was estimated as the highest price at which they indicated a positive quantity of the products^59^. In addition, the following formula was used^72,84^.

1 $${\rm{W}}{\rm{T}}{\rm{P}}( \% )=\frac{WTP\,sustainable-P\,conventional}{P\,conventional}\times 100 \%$$

where *WTP sustainable* refers to the maximum WTP of circular food products in this study, and *P conventional* denotes the price of conventional food products in each country. This formula was used to calculate the premium (in percentage terms) that respondents were willing to pay for circular food (over the conventional food in each country), addressing the issue of currency differences.

## Supplementary information

Supplementary information

## Data Availability

The data that support the findings of this study are available from the corresponding author upon reasonable request. Additional materials have been provided as part of the submission process or are included in the Supplementary Information.
