# Supplementary material for: Market potential for circular food in Europe based on consumer demand and willingness to pay
Source: NPJ Sci Food. 2025 Jul 21;9:146. doi: 10.1038/s41538-025-00475-y (PMC12280193; doi:10.1038/s41538-025-00475-y)
Supplement: Supplementary file 1 — Supplementary information [file 41538_2025_475_MOESM1_ESM.docx]

**Market Potential for Circular Food in Europe Based on Consumer Demand and Willingness to Pay**

**Contents**

[Supplementary Results 1](#_Toc191260709)

[Socio-demographic characteristics of the samples and frequency of food purchases 1](#_Toc191260710)

[Consumers’ environmental attitudes 1](#_Toc191260711)

[Summary statistics for the quantities of circular sliced pork, milk, and sliced bread demanded in six countries 2](#_Toc191260712)

[Correlation between consumers’ environmental attitude, opinions about the impact of farming systems on the environment, and opinions about the environmental sustainability of dietary patterns 2](#_Toc191260713)

[Supplementary Tables 4](#_Toc191260714)

[Supplementary Table 1 Price of sliced pork loin, milk, and bread in each case study from OECE (€) 4](#_Toc191260715)

[Supplementary Table 2 Socio-demographic characteristics of the samples 6](#_Toc191260716)

[Supplementary Table 3 Purchase frequency of general and specific food products (Pork, milk, and bread) 7](#_Toc191260717)

[Supplementary Table 4 PCA for environmental attitudes 8](#_Toc191260718)

[Supplementary Table 5 Demand for aggregate quantities of sliced pork loin from circular, organic, and conventional farming desired at different prices 9](#_Toc191260719)

[Supplementary Table 6 Demand for aggregate quantities of milk from circular, organic, and conventional farming desired at different prices 10](#_Toc191260720)

[Supplementary Table 7 Demand for aggregate quantities of sliced bread from circular, organic, and conventional farming desired at different prices 11](#_Toc191260721)

[Supplementary Table 8 Demand for circular sliced pork loin by socio-demographic and attitudinal variables 12](#_Toc191260722)

[Supplementary Table 9 Demand for circular milk by socio-demographic and attitudinal variables 14](#_Toc191260723)

[Supplementary Table 10 Demand for circular sliced bread by socio-demographic and attitudinal variables 16](#_Toc191260724)

[Supplementary Table 11 Summary statistics of the demand for quantities of circular sliced pork in six EU countries 18](#_Toc191260725)

[Supplementary Table 12 Summary statistics of the demand for quantities of circular milk in six EU countries 19](#_Toc191260726)

[Supplementary Table 13 Summary statistics of the demand for quantities of circular bread in six EU countries 20](#_Toc191260727)

[Supplementary Table 14 NB regression of circular products in Spain 21](#_Toc191260728)

[Supplementary Table 15 NB regression of circular products in Poland 22](#_Toc191260729)

[Supplementary Table 16 NB regression of circular products in Italy 23](#_Toc191260730)

[Supplementary Table 17 NB regression of circular products in Hungary 24](#_Toc191260731)

[Supplementary Table 18 NB regression of circular products in Croatia 25](#_Toc191260732)

[Supplementary Table 19 NB regression of circular products in Belgium 26](#_Toc191260733)

[Supplementary Table 20 Correlation between consumers’ environmental attitude, opinions about the impact of farming systems on the environment, and opinions about the environmental sustainability of dietary patterns 27](#_Toc191260734)

[Supplementary Figures 28](#_Toc191260735)

[Supplementary Figure 1 An example of milk in the OECE and definitions of various farming systems for milk 28](#_Toc191260736)

[Supplementary Figure 2 An example of sliced bread in the OECE and definitions of various farming systems for bread 29](#_Toc191260737)

[Supplementary Figure 3 Geographical location of the study area of this research 30](#_Toc191260738)

[Supplementary Figure 4 Observed demand for circular food products by gender in six countries 33](#_Toc191260739)

[Supplementary Figure 5 Observed demand for circular food products by age in six countries 32](#_Toc191260739)

[Supplementary Figure 6 Observed demand for circular food products by education levels in six countries 33](#_Toc191260740)

[Supplementary Figure 7 Observed demand for circular food products by environmental attitudes in six countries. 34](#_Toc191260741)

[Supplementary Figure 8 PCA results of environmental attitudes 35](#_Toc191260742)

[Supplementary Figure 9 Consumers’ environmental attitudes in each country 36](#_Toc191260743)

[Full Questionnaire 37](#_Toc191260744)

#

# Supplementary Results

## Socio-demographic characteristics of the samples and frequency of food purchases

The socio-demographic characteristics of the samples are displayed in Supplementary Table 2. The proportion of men and women was acceptable in accordance with the population distribution of each country. Nevertheless, in Poland, the proportion of females was slightly higher (59.52%). The age distribution was similar in most countries. However, a smaller proportion of people aged 35–44 in Croatia was found. The majority of respondents in all countries had completed secondary and university education and were full-time employees. In terms of stated financial situation, consumers in all countries considered it to be a little higher than the average (i.e., >5 points on a 10-point Likert scale), which could be described as a normal financial situation. Regarding the frequency of monthly income covering household expenditure, most countries had higher percentages in the “always” and “very often” categories and lower percentages in the “rarely” and “never” categories. In Italy and Hungary, the proportion of the “always” category was much higher than that of other categories. These results reveal that in all countries the majority of respondents’ income could cover their expenditure.

Analyzing the general food purchasing frequency, Supplementary Table 3 reveals consistent patterns across countries. Among all countries, the most preferred frequency was “2–3 times a week”, while the least preferred was “once a month or less” and “never”. The results of the frequency of pork (sliced pork loin) purchase (Supplementary Table 3) show that the majority of the people preferred “once a week”, while the smallest number of people preferred “daily” in most countries. Regarding the frequency of milk purchase, the results indicate that the majority of consumers bought milk “once a week” in all countries. With regard to the purchase of bread by country, the results demonstrate that bread was purchased more frequently than the other two products. In Spain and Croatia, the majority of consumers bought bread “daily”. In Poland, Italy, Hungary and Belgium, the major purchasing frequency was “2–3 times a week”.

## Consumers’ environmental attitudes

By applying Principal Component Analysis (PCA), we observed two clear dimensions in all countries (see Supplementary Table 4 and Supplementary Figure 8). The Kaiser-Meyer-Olkin test (KMO>0.75) indicated good fit and applicability of this method for the set of analyzed variables in each country. Moreover, the accumulated explained variance of the two principal components was higher than 55% in each country (Supplementary Table 4). The total variance explained by the first and second dimensions for each country was within the acceptable range of the NEP scale, thus confirming its suitability in describing environmental attitudes.

The results of consumers’ environmental attitudes are displayed in Supplementary Figure 9. Figure 9a illustrates the distribution of consumers in each country in both dimensions. We found that consumers in the second and fourth quadrants had a clear environmental attitude: consumers in the second quadrant (-ecocentric, +anthropocentric) had an anthropocentric attitude. They agreed more with anthropocentric statements and less with ecocentric statements; consumers in the fourth quadrant (+ecocentric, -anthropocentric) had an ecocentric attitude. They agreed less with statements related to anthropocentric attitudes and more with ecocentric statements. However, the environmental attitudes of consumers in the first and third quadrants were less clear and undetermined. Consumers in the first quadrant (+ecocentric, +anthropocentric) agreed with both attitude-related statements, while those in the third quadrant (-ecocentric, -anthropocentric) disagreed with both attitude-related statements. However, although respondents in these two quadrants did not have explicit environmental attitudes, their results were not exactly equal across the two potential factors, meaning that one of the two attitudes was more pronounced than the other. Our results showed that the respondents from Italy, Hungary, and Croatia were more concentrated in the fourth quadrant, highlighting their ecocentric attitudes, which meant that these consumers displayed pro-environmental behaviors and recognized the value of nature in itself and not only as a means of producing a good to satisfy their needs. They displayed a pro-environmental consumption behavior. However, consumers in Spain, Poland, and Belgium were more dispersed, with slightly more in the second quadrant, indicating that they hold anthropocentric attitudes. That is, they regarded humans as the most important component of life, and reflected an attitude related to carrying out actions that satisfy needs and achieve human well-being above everything else, and their consumption behavior was not focused on the environment.

The results suggested that an anthropocentric attitude was more pronounced in Spain, Poland, and Belgium with 54.48%, 54.62%, and 56.01%, respectively (Fig. 9b). However, in Italy, Hungary, and Croatia, the proportion of ecocentric attitudes was higher than that of anthropocentric attitudes with 51.52%, 50.91%, and 53.16%, respectively. From an overall perspective, 52.18% of the respondents exhibited an anthropocentric attitude, and 47.82% of them displayed an ecocentric attitude.

## Summary statistics for the quantities of circular sliced pork, milk, and sliced bread demanded in six countries

The summary statistics for the quantities of circular sliced pork, milk, and sliced bread demanded in six countries are displayed in Supplementary Tables 11 to 13, which were based on the results of individual consumers who indicated that they would buy at least one unit of the product, regardless of the farming system.

## Correlation between consumers’ environmental attitude, opinions about the impact of farming systems on the environment, and opinions about the environmental sustainability of dietary patterns

Results from Supplementary Table 20 reveal that ecocentric consumers in all countries were positively correlated with vegetarian, vegan and flexitarian diets, and negatively correlated with non-restricted diets. Ecocentric consumers perceived the chosen diets of vegetarian, vegan, and flexitarian to be more environmentally sustainable, while non-restricted diets were perceived as being less sustainable. Results also show that there was a positive correlation between ecocentric consumers and organic and circular farming and a negative correlation with conventional farming in five countries.

# Supplementary Tables

## **Supplementary Table 1 Price of sliced pork loin, milk, and bread in each case study from OECEs (€)**

| **Product** |  | **Pork: Sliced pork loin packs (500 g each)** | | | | | |
| --- | --- | --- | --- | --- | --- | --- | --- |
| Farming system | Purchase situation | Spain (€) | Poland (€) | Italy (€) | Hungary (€) | Croatia (€) | Belgium (€) |
| Circular | 1 | 2.50 | 1.97 | 2.80 | 2.93 | 3.09 | 4.50 |
|  | 2 | 3.50 | 2.41 | 3.40 | 3.13 | 3.55 | 5.50 |
|  | 3 | 4.50 | 2.85 | 4.20 | 3.32 | 4.02 | 6.50 |
|  | 4 | 5.50 | 3.29 | 5.00 | 3.52 | 4.48 | 7.50 |
|  | 5 | 6.50 | 3.73 | 5.80 | 3.72 | 4.95 | 8.50 |
| Conventional | 1 | 4.50 | 2.85 | 4.20 | 3.32 | 4.02 | 6.50 |
|  | 2 | 4.50 | 2.85 | 4.20 | 3.32 | 4.02 | 6.50 |
|  | 3 | 4.50 | 2.85 | 4.20 | 3.32 | 4.02 | 6.50 |
|  | 4 | 4.50 | 2.85 | 4.20 | 3.32 | 4.02 | 6.50 |
|  | 5 | 4.50 | 2.85 | 4.20 | 3.32 | 4.02 | 6.50 |
| Organic | 1 | 10.50 | 6.69 | 11.00 | 6.31 | 7.04 | 15.00 |
|  | 2 | 10.50 | 6.69 | 11.00 | 6.31 | 7.04 | 15.00 |
|  | 3 | 10.50 | 6.69 | 11.00 | 6.31 | 7.04 | 15.00 |
|  | 4 | 10.50 | 6.69 | 11.00 | 6.31 | 7.04 | 15.00 |
|  | 5 | 10.50 | 6.69 | 11.00 | 6.31 | 7.04 | 15.00 |
| **Product** |  | **Milk: Milk packs (1 liter/pack)** | | | | | |
| Farming system | Purchase situation | Spain | Poland | Italy | Hungary | Croatia | Belgium |
| Circular | 1 | 0.55 | 0.43 | 0.85 | 0.66 | 0.42 | 0.75 |
|  | 2 | 0.65 | 0.47 | 0.95 | 0.76 | 0.58 | 0.85 |
|  | 3 | 0.75 | 0.54 | 1.05 | 0.86 | 0.74 | 0.95 |
|  | 4 | 0.85 | 0.60 | 1.15 | 0.96 | 0.90 | 1.05 |
|  | 5 | 0.95 | 0.67 | 1.25 | 1.06 | 1.06 | 1.15 |
| Conventional | 1 | 0.75 | 0.54 | 1.05 | 0.86 | 0.74 | 0.95 |
|  | 2 | 0.75 | 0.54 | 1.05 | 0.86 | 0.74 | 0.95 |
|  | 3 | 0.75 | 0.54 | 1.05 | 0.86 | 0.74 | 0.95 |
|  | 4 | 0.75 | 0.54 | 1.05 | 0.86 | 0.74 | 0.95 |
|  | 5 | 0.75 | 0.54 | 1.05 | 0.86 | 0.74 | 0.95 |
| Organic | 1 | 1.35 | 1.86 | 1.65 | 1.46 | 1.79 | 1.55 |
|  | 2 | 1.35 | 1.86 | 1.65 | 1.46 | 1.79 | 1.55 |
|  | 3 | 1.35 | 1.86 | 1.65 | 1.46 | 1.79 | 1.55 |
|  | 4 | 1.35 | 1.86 | 1.65 | 1.46 | 1.79 | 1.55 |
|  | 5 | 1.35 | 1.86 | 1.65 | 1.46 | 1.79 | 1.55 |
| **Product** |  | **Bread: Sliced bread packs (450 g each)** | | | | | |
| Farming system | Purchase situation | Spain | Poland | Italy | Hungary | Croatia | Belgium |
| Circular | 1 | 0.70 | 0.71 | 0.75 | 0.86 | 0.86 | 1.35 |
|  | 2 | 0.90 | 1.04 | 0.85 | 0.96 | 1.07 | 1.45 |
|  | 3 | 1.10 | 1.37 | 0.95 | 1.06 | 1.29 | 1.55 |
|  | 4 | 1.30 | 1.70 | 1.05 | 1.16 | 1.50 | 1.65 |
|  | 5 | 1.50 | 2.03 | 1.15 | 1.26 | 1.72 | 1.75 |
| Conventional | 1 | 1.10 | 1.37 | 0.95 | 1.06 | 1.29 | 1.55 |
|  | 2 | 1.10 | 1.37 | 0.95 | 1.06 | 1.29 | 1.55 |
|  | 3 | 1.10 | 1.37 | 0.95 | 1.06 | 1.29 | 1.55 |
|  | 4 | 1.10 | 1.37 | 0.95 | 1.06 | 1.29 | 1.55 |
|  | 5 | 1.10 | 1.37 | 0.95 | 1.06 | 1.29 | 1.55 |
| Organic | 1 | 3.25 | 3.56 | 1.70 | 1.67 | 2.52 | 2.40 |
|  | 2 | 3.25 | 3.56 | 1.70 | 1.67 | 2.52 | 2.40 |
|  | 3 | 3.25 | 3.56 | 1.70 | 1.67 | 2.52 | 2.40 |
|  | 4 | 3.25 | 3.56 | 1.70 | 1.67 | 2.52 | 2.40 |
|  | 5 | 3.25 | 3.56 | 1.70 | 1.67 | 2.52 | 2.40 |

Note: At the time of data collection, participants were presented with prices from the questionnaire in the local currency of each country, and when analyzing the data, the currencies of Poland, Hungary, and Croatia were converted to euros, based on the exchange rate of March 2022. The prices shown in this table are the converted and rounded euro values.

## **Supplementary Table 2 Socio-demographic characteristics of the samples**

| **Country** | | **Spain** | **Poland** | **Italy** | **Hungary** | **Croatia** | **Belgium** |
| --- | --- | --- | --- | --- | --- | --- | --- |
| Sample Size (*n*) | | 1050 | 1040 | 755 | 988 | 506 | 950 |
|  | | **Percentage (%)** | | | | | |
| Gender | Male | 49.52 | 40.48 | 47.15 | 48.79 | 54.74 | 51.37 |
|  | Female | 50.48 | 59.52 | 52.85 | 51.21 | 45.26 | 48.63 |
| Age (years) | 18–24 | 9.43 | 21.44 | 10.99 | 10.02 | 21.34 | 10.32 |
|  | 25–34 | 15.62 | 24.62 | 21.46 | 15.38 | 28.26 | 19.05 |
|  | 35–44 | 21.52 | 13.17 | 17.75 | 21.56 | 8.50 | 18.95 |
|  | 45–54 | 21.81 | 19.52 | 15.10 | 21.86 | 23.91 | 20.42 |
|  | 55 or older | 31.62 | 21.25 | 34.70 | 31.18 | 17.99 | 31.26 |
| Education level | Not completed elementary studies | 1.81 | 0.87 | 0.53 | 4.08 | 0.40 | 2.85 |
|  | Elementary studies | 7.33 | 2.79 | 5.83 | 16.44 | 1.79 | 5.91 |
|  | Secondary studies | 45.62 | 50.38 | 51.13 | 42.62 | 55.34 | 44.20 |
|  | University studies | 45.24 | 45.96 | 42.51 | 36.86 | 42.47 | 47.04 |
| Employment status | Student | 5.69 | 9.67 | 9.17 | 5.32 | 11.76 | 5.93 |
|  | Self-employed/Business owner (full time) | 9.69 | 9.77 | 17.52 | 8.66 | 9.59 | 7.41 |
|  | Employee (full time) | 51.98 | 57.27 | 40.96 | 56.26 | 51.68 | 52.98 |
|  | A homemaker | 6.55 | 6.4 | 4.93 | 5.84 | 4.05 | 5.2 |
|  | On sick leave | 0.93 | 2.07 | 1.22 | 1.16 | 3.07 | 2.56 |
|  | Unemployed | 12.36 | 4.48 | 10.5 | 7.72 | 11.56 | 5.41 |
|  | Retired | 9.22 | 8.61 | 14.07 | 12.82 | 7.02 | 14.27 |
|  | Unable to work | 3.58 | 1.73 | 1.63 | 2.22 | 1.27 | 6.24 |
| Monthly income covers their household expenditure | Always | 39.14 | 36.73 | 52.45 | 44.66 | 28.66 | 29.32 |
|  | Very Often | 28.10 | 31.15 | 25.43 | 24.13 | 29.25 | 29.22 |
|  | Sometimes | 23.62 | 22.21 | 16.82 | 19.15 | 24.90 | 29.96 |
|  | Rarely | 7.04 | 7.12 | 4.11 | 6.77 | 13.24 | 8.65 |
|  | Never | 2.10 | 2.79 | 1.19 | 5.29 | 3.95 | 2.85 |
|  | **Scale** | **Mean (SD)** | | | | | |
| Stated current financial situation | 10-point Likert scale (1=very difficult, 10=very good) | 5.70 (2.02) | 5.83 (2.04) | 5.86 (1.95) | 5.43 (2.15) | 5.64 (2.19) | 5.85 (2.09) |

## **Supplementary** **Table 3 Purchase frequency of general and specific food products (Pork, milk, and bread)**

| **Country** | | **Spain** | **Poland** | **Italy** | **Hungary** | **Croatia** | **Belgium** |
| --- | --- | --- | --- | --- | --- | --- | --- |
| Sample Size (*n*) | | 1050 | 1040 | 755 | 988 | 506 | 950 |
| **Percentage (%)** | | | | | | | |
| Food (in general) Purchase Frequency | Daily | 16.57 | 30.58 | 13.67 | 20.55 | 24.51 | 11.86 |
|  | 2–3 times a week | **37.62** | **46.54** | **44.79** | **45.65** | **42.89** | **39.30** |
|  | Once a week | 31.81 | 17.12 | 32.75 | 26.42 | 23.91 | 37.18 |
|  | 2–3 times per month | 10.29 | 4.42 | 7.04 | 4.66 | 4.55 | 8.26 |
|  | Once a month or less | 3.52 | 1.15 | 1.62 | 2.13 | 3.36 | 2.44 |
|  | Never | 0.19 | 0.19 | 0.13 | 0.59 | 0.78 | 0.96 |
| Pork Purchase Frequency | Daily | 3.90 | 3.65 | 2.57 | 2.53 | 3.36 | 3.07 |
|  | 2–3 times a week | 18.48 | 19.33 | 8.80 | 8.91 | 18.38 | 13.67 |
|  | Once a week | **42.67** | **38.08** | **36.94** | **34.31** | **37.55** | **34.22** |
|  | 2–3 times per month | 20.48 | 22.02 | 24.76 | 28.14 | 18.97 | 22.35 |
|  | Once a month or less | 10.76 | 11.06 | 18.67 | 18.52 | 16.60 | 15.57 |
|  | Never | 3.71 | 5.86 | 8.26 | 7.59 | 5.14 | 11.12 |
| Milk Purchase Frequency | Daily | 8.67 | 10.00 | 8.66 | 6.98 | 15.02 | 9.32 |
|  | 2–3 times a week | 20.00 | 30.38 | 25.30 | 21.56 | 28.85 | 10.91 |
|  | Once a week | **37.90** | **36.63** | **36.94** | **35.02** | **30.43** | **31.89** |
|  | 2–3 times per month | 19.52 | 13.46 | 12.31 | 16.70 | 12.06 | 21.08 |
|  | Once a month or less | 10.38 | 5.29 | 8.80 | 11.44 | 9.68 | 19.49 |
|  | Never | 3.53 | 4.24 | 7.99 | 8.30 | 3.96 | 7.31 |
| Bread Purchase Frequency | Daily | **53.90** | 33.85 | 29.99 | 18.83 | **48.42** | 20.55 |
|  | 2–3 times a week | 19.71 | **41.15** | **32.56** | **45.45** | 28.85 | **34.22** |
|  | Once a week | 15.71 | 13.85 | 23.74 | 18.93 | 10.87 | 26.27 |
|  | 2–3 times per month | 5.33 | 4.23 | 6.38 | 6.88 | 4.74 | 8.05 |
|  | Once a month or less | 3.52 | 3.46 | 5.29 | 4.45 | 3.95 | 5.19 |
|  | Never | 1.83 | 3.46 | 2.04 | 5.46 | 3.17 | 5.72 |

## **Supplementary Table 4 PCA for environmental attitudes**

|  | **Spain** | | **Croatia** | | **Hungary** | | **Italy** | | **Poland** | | **Belgium** | |
| --- | --- | --- | --- | --- | --- | --- | --- | --- | --- | --- | --- | --- |
|  | **Component** | | **Component** | | **Component** | | **Component** | | **Component** | | **Component** | |
|  | **1** | **2** | **1** | **2** | **1** | **2** | **1** | **2** | **1** | **2** | **1** | **2** |
| Item 1. The balance of nature is strong enough to deal with the impact caused by economic development | -0.165 | 0.744 | -0.252 | 0.672 | -0.305 | 0.662 | -0.297 | 0.696 | -0.137 | 0.728 | -0.243 | 0.724 |
| Item 2. Over time, humans can learn how nature works to be able to control it | 0.095 | 0.781 | 0.139 | 0.730 | 0.106 | 0.844 | 0.062 | 0.800 | 0.205 | 0.760 | 0.183 | 0.723 |
| Item 3. Human ingenuity will ensure that we do not make the earth an uninhabitable place | 0.074 | 0.776 | 0.067 | 0.737 | 0.215 | 0.787 | 0.083 | 0.786 | 0.149 | 0.686 | 0.074 | 0.772 |
| Item 4. Humans have the right to modify the environment to adapt it to their needs | -0.236 | 0.703 | -0.396 | 0.586 | -0.380 | 0.563 | -0.377 | 0.660 | -0.229 | 0.668 | -0.244 | 0.711 |
| Item 5. Plants and animals have as much right to exist as humans | 0.805 | -0.045 | 0.748 | -0.056 | 0.754 | -0.033 | 0.740 | -0.043 | 0.782 | -0.003 | 0.741 | -0.029 |
| Item 6. The balance of nature is very delicate and easily alterable | 0.847 | 0.056 | 0.776 | -0.008 | 0.830 | -0.029 | 0.818 | -0.093 | 0.663 | 0.100 | 0.662 | 0.006 |
| Item 7. If things continue as they are, we will soon face a major ecological catastrophe | 0.815 | -0.081 | 0.777 | -0.115 | 0.827 | -0.142 | 0.807 | -0.142 | 0.822 | -0.137 | 0.770 | -0.198 |
| Item 8. Despite our special abilities, humans are still dependent on the laws of nature | 0.801 | 0.020 | 0.714 | -0.020 | 0.841 | 0.058 | 0.809 | -0.045 | 0.810 | 0.008 | 0.765 | 0.025 |
| Explained variance by component (%) | 36.60 | 26.42 | 31.49 | 23.61 | 36.80 | 26.42 | 34.55 | 27.63 | 31.48 | 25.68 | 32.17 | 24.23 |
| Total explained variance | 63.02% | | 55.10% | | 63.22% | | 62.18% | | 57.16% | | 56.40% | |
| (KMO) Kaiser-Meyer-Olkin Test | 0.794 | | 0.777 | | 0.778 | | 0.812 | | 0.758 | | 0.753 | |
| Bartlett Test Chi-square | 2656.45 | | 816.35 | | 2655.82 | | 1835.27 | | 1923.62 | | 1642.43 | |
| *p*-value | <0.001 | | <0.001 | | <0.001 | | <0.001 | | <0.001 | | <0.001 | |

## **Supplementary Table 5** Demand for aggregate quantities **of sliced pork loin from circular, organic, and conventional farming desired at different prices**

| Sliced pork loin | | | | | | | | | | | | | | | | | | | | |
| --- | --- | --- | --- | --- | --- | --- | --- | --- | --- | --- | --- | --- | --- | --- | --- | --- | --- | --- | --- | --- |
| Spain (n=1050) | | | | | | | | | |  | Poland (n=1040) | | | | | | | | | |
| Circular | | | Organic | | | Conventional | | |  |  | Circular | | | Organic | | | Conventional | | |  |
| Price | Quantity | % | Price | Quantity | % | Price | Quantity | % | Total |  | Price | Quantity | % | Price | Quantity | % | Price | Quantity | % | Total |
| 2.50 | 1760 | 43.5% | 10.50 | 904 | 22.3% | 4.50 | 1382 | 34.2% | 4046 |  | 1.97 | 1639 | 42.3% | 6.69 | 1011 | 26.1% | 2.85 | 1228 | 31.6% | 3878 |
| 3.50 | 1698 | 43.1% | 10.50 | 910 | 23.1% | 4.50 | 1335 | 33.8% | 3943 |  | 2.41 | 1578 | 40.5% | 6.69 | 1039 | 26.6% | 2.85 | 1282 | 32.9% | 3899 |
| 4.50 | 1587 | 40.5% | 10.50 | 898 | 22.9% | 4.50 | 1430 | 36.6% | 3915 |  | 2.85 | 1446 | 38.0% | 6.69 | 981 | 25.8% | 2.85 | 1376 | 36.2% | 3803 |
| 5.50 | 1277 | 34.5% | 10.50 | 900 | 24.3% | 4.50 | 1520 | 41.2% | 3697 |  | 3.29 | 1243 | 33.8% | 6.69 | 1022 | 27.8% | 2.85 | 1413 | 38.4% | 3678 |
| 6.50 | 1123 | 30.2% | 10.50 | 916 | 24.6% | 4.50 | 1681 | 45.2% | 3720 |  | 3.73 | 1074 | 29.7% | 6.69 | 1028 | 28.4% | 2.85 | 1518 | 41.9% | 3620 |
| Italy (n=755) | | | | | | | | | |  | Hungary (n=988) | | | | | | | | | |
| Circular | | | Organic | | | Conventional | | |  |  | Circular | | | Organic | | | Conventional | | |  |
| Price | Quantity | % | Price | Quantity | % | Price | Quantity | % | Total |  | Price | Quantity | % | Price | Quantity | % | Price | Quantity | % | Total |
| 2.80 | 1035 | 48.9% | 11.00 | 516 | 24.4% | 4.20 | 565 | 26.7% | 2116 |  | 2.93 | 1624 | 47.0% | 6.31 | 495 | 14.3% | 3.32 | 1335 | 38.7% | 3454 |
| 3.40 | 992 | 47.0% | 11.00 | 530 | 25.1% | 4.20 | 588 | 27.9% | 2110 |  | 3.13 | 1594 | 45.6% | 6.31 | 520 | 14.9% | 3.32 | 1380 | 39.5% | 3494 |
| 4.20 | 947 | 45.8% | 11.00 | 507 | 24.5% | 4.20 | 612 | 29.7% | 2066 |  | 3.32 | 1580 | 43.7% | 6.31 | 561 | 15.5% | 3.32 | 1473 | 40.8% | 3614 |
| 5.00 | 808 | 40.2% | 11.00 | 516 | 25.7% | 4.20 | 684 | 34.1% | 2008 |  | 3.52 | 1295 | 37.4% | 6.31 | 523 | 15.1% | 3.32 | 1644 | 47.5% | 3462 |
| 5.80 | 713 | 37.1% | 11.00 | 521 | 27.1% | 4.20 | 690 | 35.8% | 1924 |  | 3.72 | 1084 | 31.8% | 6.31 | 531 | 15.6% | 3.32 | 1799 | 52.6% | 3414 |
| Croatia (n=506) | | | | | | | | | |  | Belgium (n=950) | | | | | | | | | |
| Circular | | | Organic | | | Conventional | | |  |  | Circular | | | Organic | | | Conventional | | |  |
| Price | Quantity | % | Price | Quantity | % | Price | Quantity | % | Total |  | Price | Quantity | % | Price | Quantity | % | Price | Quantity | % | Total |
| 3.09 | 882 | 43.9% | 7.04 | 502 | 25.0% | 4.02 | 627 | 31.1% | 2011 |  | 4.50 | 1094 | 44.0% | 15.00 | 512 | 20.6% | 6.50 | 878 | 35.4% | 2484 |
| 3.55 | 857 | 43.3% | 7.04 | 497 | 25.1% | 4.02 | 623 | 31.6% | 1977 |  | 5.50 | 1060 | 43.1% | 15.00 | 494 | 20.1% | 6.50 | 906 | 36.8% | 2460 |
| 4.02 | 748 | 38.2% | 7.04 | 543 | 27.8% | 4.02 | 665 | 34.0% | 1956 |  | 6.50 | 1007 | 40.7% | 15.00 | 511 | 20.7% | 6.50 | 954 | 38.6% | 2472 |
| 4.48 | 602 | 31.8% | 7.04 | 530 | 28.0% | 4.02 | 760 | 40.2% | 1892 |  | 7.50 | 807 | 33.8% | 15.00 | 517 | 21.6% | 6.50 | 1067 | 44.6% | 2391 |
| 4.95 | 524 | 27.4% | 7.04 | 558 | 29.1% | 4.02 | 833 | 43.5% | 1915 |  | 8.50 | 724 | 30.3% | 15.00 | 531 | 22.2% | 6.50 | 1135 | 47.5% | 2390 |

Note: All prices are in Euros.

## **Supplementary Table 6** Demand for aggregate quantities **of milk from circular, organic, and conventional farming desired at different prices**

| Milk | | | | | | | | | | | | | | | | | | | | |
| --- | --- | --- | --- | --- | --- | --- | --- | --- | --- | --- | --- | --- | --- | --- | --- | --- | --- | --- | --- | --- |
| Spain (n=1050) | | | | | | | | | |  | Poland (n=1040) | | | | | | | | | |
| Circular | | | Organic | | | Conventional | | |  |  | Circular | | | Organic | | | Conventional | | |  |
| Price | Quantity | % | Price | Quantity | % | Price | Quantity | % | Total |  | Price | Quantity | % | Price | Quantity | % | Price | Quantity | % | Total |
| 0.55 | 2772 | 44.4% | 1.35 | 1325 | 21.2% | 0.75 | 2145 | 34.4% | 6242 |  | 0.43 | 2369 | 44.1% | 1.86 | 1137 | 21.2% | 0.54 | 1862 | 34.7% | 5368 |
| 0.65 | 2623 | 42.4% | 1.35 | 1380 | 22.3% | 0.75 | 2177 | 35.3% | 6180 |  | 0.47 | 2163 | 41.1% | 1.86 | 1158 | 22.0% | 0.54 | 1941 | 36.9% | 5262 |
| 0.75 | 2445 | 39.7% | 1.35 | 1359 | 22.1% | 0.75 | 2353 | 38.2% | 6157 |  | 0.54 | 2026 | 38.8% | 1.86 | 1194 | 22.9% | 0.54 | 1998 | 38.3% | 5218 |
| 0.85 | 1925 | 31.8% | 1.35 | 1426 | 23.6% | 0.75 | 2698 | 44.6% | 6049 |  | 0.60 | 1618 | 32.1% | 1.86 | 1253 | 24.9% | 0.54 | 2163 | 43.0% | 5034 |
| 0.95 | 1644 | 27.7% | 1.35 | 1452 | 24.5% | 0.75 | 2834 | 47.8% | 5930 |  | 0.67 | 1432 | 28.7% | 1.86 | 1256 | 25.2% | 0.54 | 2304 | 46.1% | 4992 |
| Italy (n=755) | | | | | | | | | |  | Hungary (n=988) | | | | | | | | | |
| Circular | | | Organic | | | Conventional | | |  |  | Circular | | | Organic | | | Conventional | | |  |
| Price | Quantity | % | Price | Quantity | % | Price | Quantity | % | Total |  | Price | Quantity | % | Price | Quantity | % | Price | Quantity | % | Total |
| 0.85 | 1235 | 49.2% | 1.65 | 591 | 23.6% | 1.05 | 682 | 27.2% | 2508 |  | 0.66 | 2340 | 59.8% | 1.46 | 383 | 9.8% | 0.86 | 1192 | 30.4% | 3915 |
| 0.95 | 1175 | 46.8% | 1.65 | 622 | 24.8% | 1.05 | 714 | 28.4% | 2511 |  | 0.76 | 2243 | 58.2% | 1.46 | 402 | 10.4% | 0.86 | 1207 | 31.4% | 3852 |
| 1.05 | 1065 | 43.5% | 1.65 | 629 | 25.7% | 1.05 | 753 | 30.8% | 2447 |  | 0.86 | 1829 | 49.3% | 1.46 | 381 | 10.3% | 0.86 | 1503 | 40.4% | 3713 |
| 1.15 | 866 | 37.0% | 1.65 | 618 | 26.4% | 1.05 | 859 | 36.6% | 2343 |  | 0.96 | 1101 | 31.0% | 1.46 | 393 | 11.1% | 0.86 | 2056 | 57.9% | 3550 |
| 1.25 | 753 | 32.3% | 1.65 | 638 | 27.4% | 1.05 | 940 | 40.3% | 2331 |  | 1.06 | 886 | 24.8% | 1.46 | 449 | 12.5% | 0.86 | 2244 | 62.7% | 3579 |
| Croatia (n=506) | | | | | | | | | |  | Belgium (n=950) | | | | | | | | | |
| Circular | | | Organic | | | Conventional | | |  |  | Circular | | | Organic | | | Conventional | | |  |
| Price | Quantity | % | Price | Quantity | % | Price | Quantity | % | Total |  | Price | Quantity | % | Price | Quantity | % | Price | Quantity | % | Total |
| 0.42 | 1753 | 51.7% | 1.79 | 696 | 20.5% | 0.74 | 944 | 27.8% | 3393 |  | 0.75 | 1877 | 46.0% | 1.55 | 811 | 19.9% | 0.95 | 1396 | 34.1% | 4084 |
| 0.58 | 1559 | 46.5% | 1.79 | 720 | 21.5% | 0.74 | 1076 | 32.0% | 3355 |  | 0.85 | 1794 | 44.5% | 1.55 | 819 | 20.3% | 0.95 | 1417 | 35.2% | 4030 |
| 0.74 | 1238 | 39.5% | 1.79 | 718 | 22.9% | 0.74 | 1178 | 37.6% | 3134 |  | 0.95 | 1700 | 41.1% | 1.55 | 884 | 21.4% | 0.95 | 1550 | 37.5% | 4134 |
| 0.90 | 829 | 27.7% | 1.79 | 716 | 23.9% | 0.74 | 1447 | 48.4% | 2992 |  | 1.05 | 1330 | 33.5% | 1.55 | 909 | 22.9% | 0.95 | 1727 | 43.6% | 3966 |
| 1.06 | 698 | 21.9% | 1.79 | 841 | 26.4% | 0.74 | 1641 | 51.7% | 3180 |  | 1.15 | 1107 | 28.1% | 1.55 | 929 | 23.6% | 0.95 | 1901 | 48.3% | 3937 |

Note: All prices are in Euros.

## **Supplementary Table 7** Demand for aggregate quantities **of sliced bread from circular, organic, and conventional farming desired at different prices**

| Sliced bread | | | | | | | | | | | | | | | | | | | | |
| --- | --- | --- | --- | --- | --- | --- | --- | --- | --- | --- | --- | --- | --- | --- | --- | --- | --- | --- | --- | --- |
| Spain (n=1050) | | | | | | | | | |  | Poland (n=1040) | | | | | | | | | |
| Circular | | | Organic | | | Conventional | | |  |  | Circular | | | Organic | | | Conventional | | |  |
| Price | Quantity | % | Price | Quantity | % | Price | Quantity | % | Total |  | Price | Quantity | % | Price | Quantity | % | Price | Quantity | % | Total |
| 0.70 | 1483 | 45.1% | 3.25 | 691 | 21.0% | 1.10 | 1114 | 33.9% | 3288 |  | 0.71 | 1591 | 48.9% | 3.56 | 698 | 21.5% | 1.37 | 963 | 29.6% | 3252 |
| 0.90 | 1472 | 43.9% | 3.25 | 718 | 21.4% | 1.10 | 1164 | 34.7% | 3354 |  | 1.04 | 1427 | 44.8% | 3.56 | 708 | 22.2% | 1.37 | 1053 | 33.0% | 3188 |
| 1.10 | 1287 | 38.8% | 3.25 | 750 | 22.6% | 1.10 | 1279 | 38.6% | 3316 |  | 1.37 | 1173 | 37.9% | 3.56 | 741 | 24.0% | 1.37 | 1177 | 38.1% | 3091 |
| 1.30 | 1101 | 34.0% | 3.25 | 750 | 23.1% | 1.10 | 1392 | 42.9% | 3243 |  | 1.70 | 938 | 31.9% | 3.56 | 718 | 24.4% | 1.37 | 1287 | 43.7% | 2943 |
| 1.50 | 1008 | 31.4% | 3.25 | 755 | 23.5% | 1.10 | 1447 | 45.1% | 3210 |  | 2.03 | 841 | 27.7% | 3.56 | 777 | 25.6% | 1.37 | 1414 | 46.7% | 3032 |
| Italy (n=755) | | | | | | | | | |  | Hungary (n=988) | | | | | | | | | |
| Circular | | | Organic | | | Conventional | | |  |  | Circular | | | Organic | | | Conventional | | |  |
| Price | Quantity | % | Price | Quantity | % | Price | Quantity | % | Total |  | Price | Quantity | % | Price | Quantity | % | Price | Quantity | % | Total |
| 0.75 | 827 | 46.3% | 1.70 | 449 | 25.1% | 0.95 | 512 | 28.6% | 1788 |  | 0.86 | 1111 | 52.3% | 1.67 | 257 | 12.1% | 1.06 | 758 | 35.6% | 2126 |
| 0.85 | 806 | 44.4% | 1.70 | 464 | 25.6% | 0.95 | 544 | 30.0% | 1814 |  | 0.96 | 1077 | 52.4% | 1.67 | 242 | 11.8% | 1.06 | 736 | 35.8% | 2055 |
| 0.95 | 761 | 41.4% | 1.70 | 509 | 27.7% | 0.95 | 569 | 30.9% | 1839 |  | 1.06 | 989 | 46.5% | 1.67 | 265 | 12.5% | 1.06 | 871 | 41.0% | 2125 |
| 1.05 | 619 | 36.4% | 1.70 | 479 | 28.2% | 0.95 | 602 | 35.4% | 1700 |  | 1.16 | 562 | 28.8% | 1.67 | 274 | 14.0% | 1.06 | 1118 | 57.2% | 1954 |
| 1.15 | 588 | 34.4% | 1.70 | 482 | 28.2% | 0.95 | 637 | 37.4% | 1707 |  | 1.26 | 492 | 25.0% | 1.67 | 288 | 14.7% | 1.06 | 1185 | 60.3% | 1965 |
| Croatia (n=506) | | | | | | | | | |  | Belgium (n=950) | | | | | | | | | |
| Circular | | | Organic | | | Conventional | | |  |  | Circular | | | Organic | | | Conventional | | |  |
| Price | Quantity | % | Price | Quantity | % | Price | Quantity | % | Total |  | Price | Quantity | % | Price | Quantity | % | Price | Quantity | % | Total |
| 0.86 | 576 | 43.1% | 2.52 | 331 | 24.8% | 1.29 | 430 | 32.1% | 1337 |  | 1.35 | 933 | 29.9% | 2.40 | 916 | 29.4% | 1.55 | 1267 | 40.7% | 3116 |
| 1.07 | 525 | 41.5% | 2.52 | 290 | 22.9% | 1.29 | 451 | 35.6% | 1266 |  | 1.45 | 919 | 41.6% | 2.40 | 485 | 21.9% | 1.55 | 806 | 36.5% | 2210 |
| 1.29 | 484 | 36.9% | 2.52 | 335 | 25.6% | 1.29 | 491 | 37.5% | 1310 |  | 1.55 | 867 | 37.6% | 2.40 | 509 | 22.1% | 1.55 | 930 | 40.3% | 2306 |
| 1.50 | 362 | 28.5% | 2.52 | 316 | 24.9% | 1.29 | 591 | 46.6% | 1269 |  | 1.65 | 698 | 31.9% | 2.40 | 493 | 22.5% | 1.55 | 997 | 45.6% | 2188 |
| 1.72 | 351 | 26.7% | 2.52 | 331 | 25.2% | 1.29 | 633 | 48.1% | 1315 |  | 1.75 | 626 | 28.2% | 2.40 | 502 | 22.6% | 1.55 | 1090 | 49.2% | 2218 |

Note: All prices are in Euros.

## **Supplementary Table 8** Demand for circular **sliced pork loin by socio-demographic and attitudinal variables**

| **Circular sliced pork loin** | | | | | | | | | |
| --- | --- | --- | --- | --- | --- | --- | --- | --- | --- |
| **Spain (n=1050)** | | | | | | | | | |
|  | Gender | | Age (years) | | | Education levels | | Environmental attitudes | |
| Price | Male | Female | 18–34 | 35–54 | ≥55 | Below university | University | Ecocentric | Anthropocentric |
| 2.50 | 883 | 877 | 530 | 794 | 436 | 903 | 857 | 844 | 916 |
| 3.50 | 851 | 847 | 543 | 746 | 409 | 900 | 798 | 813 | 885 |
| 4.50 | 783 | 804 | 525 | 703 | 359 | 812 | 775 | 716 | 871 |
| 5.50 | 627 | 650 | 420 | 584 | 273 | 649 | 628 | 535 | 742 |
| 6.50 | 547 | 576 | 395 | 531 | 197 | 579 | 544 | 403 | 720 |
| **Poland (n=1040)** | | | | | | | | | |
|  | Gender | | Age (years) | | | Education levels | | Environmental attitudes | |
| Price | Male | Female | 18–34 | 35–54 | ≥55 | Below university | University | Ecocentric | Anthropocentric |
| 1.97 | 754 | 885 | 753 | 539 | 347 | 885 | 754 | 691 | 948 |
| 2.41 | 718 | 860 | 718 | 522 | 338 | 837 | 741 | 624 | 954 |
| 2.85 | 663 | 783 | 648 | 467 | 331 | 752 | 694 | 552 | 894 |
| 3.29 | 577 | 666 | 622 | 364 | 257 | 641 | 602 | 420 | 823 |
| 3.73 | 527 | 547 | 529 | 325 | 220 | 564 | 510 | 344 | 730 |
| **Italy (n=755)** | | | | | | | | | |
|  | Gender | | Age (years) | | | Education levels | | Environmental attitudes | |
| Price | Male | Female | 18–34 | 35–54 | ≥55 | Below university | University | Ecocentric | Anthropocentric |
| 2.80 | 540 | 495 | 401 | 343 | 291 | 588 | 447 | 481 | 554 |
| 3.40 | 503 | 489 | 386 | 322 | 284 | 585 | 407 | 454 | 538 |
| 4.20 | 499 | 448 | 364 | 299 | 284 | 545 | 402 | 435 | 512 |
| 5.00 | 412 | 396 | 335 | 236 | 237 | 464 | 344 | 352 | 456 |
| 5.80 | 351 | 362 | 284 | 214 | 215 | 405 | 308 | 320 | 393 |
| **Hungary (n=988)** | | | | | | | | | |
|  | Gender | | Age (years) | | | Education levels | | Environmental attitudes | |
| Price | Male | Female | 18–34 | 35–54 | ≥55 | Below university | University | Ecocentric | Anthropocentric |
| 2.93 | 892 | 732 | 367 | 736 | 521 | 906 | 718 | 921 | 703 |
| 3.13 | 875 | 719 | 363 | 741 | 490 | 896 | 698 | 880 | 714 |
| 3.32 | 853 | 727 | 384 | 708 | 488 | 891 | 689 | 866 | 714 |
| 3.52 | 621 | 674 | 292 | 580 | 423 | 746 | 549 | 697 | 598 |
| 3.72 | 500 | 584 | 275 | 469 | 340 | 662 | 422 | 537 | 547 |
| **Croatia (n=506)** | | | | | | | | | |
|  | Gender | | Age (years) | | | Education levels | | Environmental attitudes | |
| Price | Male | Female | 18–34 | 35–54 | ≥55 | Below university | University | Ecocentric | Anthropocentric |
| 3.09 | 498 | 384 | 463 | 286 | 133 | 530 | 352 | 476 | 406 |
| 3.55 | 498 | 359 | 451 | 281 | 125 | 498 | 359 | 441 | 416 |
| 4.02 | 451 | 297 | 407 | 236 | 105 | 424 | 324 | 373 | 375 |
| 4.48 | 367 | 235 | 328 | 188 | 86 | 347 | 255 | 288 | 314 |
| 4.95 | 313 | 211 | 310 | 147 | 67 | 303 | 221 | 219 | 305 |
| **Belgium (n=950)** | | | | | | | | | |
|  | Gender | | Age (years) | | | Education levels | | Environmental attitudes | |
| Price | Male | Female | 18–34 | 35–54 | ≥55 | Below university | University | Ecocentric | Anthropocentric |
| 4.50 | 654 | 440 | 348 | 453 | 293 | 538 | 556 | 502 | 592 |
| 5.50 | 621 | 439 | 349 | 414 | 297 | 521 | 539 | 482 | 578 |
| 6.50 | 587 | 420 | 339 | 397 | 271 | 475 | 532 | 447 | 560 |
| 7.50 | 483 | 324 | 293 | 314 | 200 | 372 | 435 | 347 | 460 |
| 8.50 | 428 | 296 | 267 | 288 | 169 | 356 | 368 | 296 | 428 |

Note: All prices are in Euros.

## **Supplementary Table 9** Demand for circular **milk by socio-demographic and attitudinal variables**

| **Circular milk** | | | | | | | | | |
| --- | --- | --- | --- | --- | --- | --- | --- | --- | --- |
| **Spain (n=1050)** | | | | | | | | | |
|  | Gender | | Age (years) | | | Education levels | | Environmental attitudes | |
| Price | Male | Female | 18–34 | 35–54 | ≥55 | Below university | University | Ecocentric | Anthropocentric |
| 0.55 | 1389 | 1383 | 828 | 1188 | 756 | 1387 | 1385 | 1476 | 1296 |
| 0.65 | 1339 | 1284 | 800 | 1120 | 703 | 1336 | 1287 | 1367 | 1256 |
| 0.75 | 1185 | 1260 | 799 | 1029 | 617 | 1211 | 1234 | 1286 | 1159 |
| 0.85 | 923 | 1002 | 679 | 812 | 434 | 963 | 962 | 925 | 1000 |
| 0.95 | 795 | 849 | 605 | 723 | 316 | 833 | 811 | 706 | 938 |
| **Poland (n=1040)** | | | | | | | | | |
|  | Gender | | Age (years) | | | Education levels | | Environmental attitudes | |
| Price | Male | Female | 18–34 | 35–54 | ≥55 | Below university | University | Ecocentric | Anthropocentric |
| 0.43 | 1102 | 1267 | 1213 | 766 | 390 | 1205 | 1164 | 1030 | 1339 |
| 0.47 | 1040 | 1123 | 1118 | 661 | 384 | 1171 | 992 | 939 | 1224 |
| 0.54 | 939 | 1087 | 1043 | 608 | 375 | 1097 | 929 | 833 | 1193 |
| 0.60 | 761 | 857 | 859 | 465 | 294 | 858 | 760 | 636 | 982 |
| 0.67 | 682 | 750 | 765 | 434 | 233 | 760 | 672 | 497 | 935 |
| **Italy (n=755)** | | | | | | | | | |
|  | Gender | | Age (years) | | | Education levels | | Environmental attitudes | |
| Price | Male | Female | 18–34 | 35–54 | ≥55 | Below university | University | Ecocentric | Anthropocentric |
| 0.85 | 676 | 559 | 469 | 386 | 380 | 643 | 592 | 634 | 601 |
| 0.95 | 622 | 553 | 425 | 364 | 386 | 613 | 562 | 591 | 584 |
| 1.05 | 563 | 502 | 399 | 308 | 358 | 569 | 496 | 537 | 528 |
| 1.15 | 452 | 414 | 306 | 261 | 299 | 442 | 424 | 430 | 436 |
| 1.25 | 382 | 371 | 273 | 227 | 253 | 377 | 376 | 360 | 393 |
| **Hungary (n=988)** | | | | | | | | | |
|  | Gender | | Age (years) | | | Education levels | | Environmental attitude | |
| Price | Male | Female | 18–34 | 35–54 | ≥55 | Below university | University | Ecocentric | Anthropocentric |
| 0.66 | 1347 | 993 | 561 | 1136 | 643 | 1269 | 1071 | 1443 | 897 |
| 0.76 | 1271 | 972 | 532 | 1113 | 598 | 1209 | 1034 | 1356 | 887 |
| 0.86 | 1010 | 819 | 418 | 896 | 515 | 933 | 896 | 1159 | 670 |
| 0.96 | 510 | 591 | 292 | 511 | 298 | 614 | 487 | 619 | 482 |
| 1.06 | 416 | 470 | 241 | 408 | 237 | 519 | 367 | 458 | 428 |
| **Croatia (n=506)** | | | | | | | | | |
|  | Gender | | Age (years) | | | Education levels | | Environmental attitudes | |
| Price | Male | Female | 18–34 | 35–54 | ≥55 | Below university | University | Ecocentric | Anthropocentric |
| 0.42 | 906 | 847 | 818 | 617 | 318 | 1029 | 724 | 953 | 800 |
| 0.58 | 806 | 753 | 763 | 542 | 254 | 929 | 630 | 829 | 730 |
| 0.74 | 711 | 527 | 611 | 414 | 213 | 739 | 499 | 624 | 614 |
| 0.90 | 525 | 304 | 458 | 247 | 124 | 437 | 392 | 321 | 508 |
| 1.06 | 449 | 249 | 422 | 180 | 96 | 370 | 328 | 197 | 501 |
| **Belgium (n=950)** | | | | | | | | | |
|  | Gender | | Age (years) | | | Education levels | | Environmental attitudes | |
| Price | Male | Female | 18–34 | 35–54 | ≥55 | Below university | University | Ecocentric | Anthropocentric |
| 0.75 | 1097 | 780 | 670 | 790 | 417 | 856 | 1021 | 955 | 922 |
| 0.85 | 1050 | 744 | 637 | 748 | 409 | 818 | 976 | 908 | 886 |
| 0.95 | 1007 | 693 | 622 | 699 | 379 | 762 | 938 | 859 | 841 |
| 1.05 | 780 | 550 | 568 | 519 | 243 | 582 | 748 | 655 | 675 |
| 1.15 | 660 | 447 | 458 | 439 | 210 | 550 | 557 | 516 | 591 |

Note: All prices are in Euros.

## **Supplementary Table 10** Demand for circular **sliced bread by socio-demographic and attitudinal variables**

| **Circular sliced bread** | | | | | | | | | |
| --- | --- | --- | --- | --- | --- | --- | --- | --- | --- |
| **Spain (n=1050)** | | | | | | | | | |
|  | Gender | | Age (years) | | | Education levels | | Environmental attitudes | |
| Price | Male | Female | 18–34 | 35–54 | ≥55 | Below university | University | Ecocentric | Anthropocentric |
| 0.70 | 692 | 791 | 518 | 660 | 305 | 743 | 740 | 670 | 813 |
| 0.90 | 697 | 775 | 516 | 673 | 283 | 732 | 740 | 643 | 829 |
| 1.10 | 581 | 706 | 466 | 576 | 245 | 646 | 641 | 546 | 741 |
| 1.30 | 515 | 586 | 425 | 513 | 163 | 552 | 549 | 420 | 681 |
| 1.50 | 476 | 532 | 397 | 470 | 141 | 503 | 505 | 364 | 644 |
| **Poland (n=1040)** | | | | | | | | | |
|  | Gender | | Age (years) | | | Education levels | | Environmental attitudes | |
| Price | Male | Female | 18–34 | 35–54 | ≥55 | Below university | University | Ecocentric | Anthropocentric |
| 0.71 | 660 | 931 | 801 | 463 | 327 | 903 | 688 | 639 | 952 |
| 1.04 | 622 | 805 | 699 | 415 | 313 | 811 | 616 | 580 | 847 |
| 1.37 | 499 | 674 | 580 | 328 | 265 | 648 | 525 | 454 | 719 |
| 1.70 | 435 | 503 | 488 | 241 | 209 | 506 | 432 | 285 | 653 |
| 2.03 | 398 | 443 | 461 | 184 | 196 | 452 | 389 | 223 | 618 |
| **Italy (n=755)** | | | | | | | | | |
|  | Gender | | Age (years) | | | Education levels | | Environmental attitudes | |
| Price | Male | Female | 18–34 | 35–54 | ≥55 | Below university | University | Ecocentric | Anthropocentric |
| 0.75 | 417 | 410 | 325 | 300 | 202 | 455 | 372 | 402 | 425 |
| 0.85 | 407 | 399 | 329 | 279 | 198 | 453 | 353 | 390 | 416 |
| 0.95 | 373 | 388 | 294 | 270 | 197 | 429 | 332 | 372 | 389 |
| 1.05 | 302 | 317 | 247 | 211 | 161 | 346 | 273 | 282 | 337 |
| 1.15 | 284 | 304 | 233 | 207 | 148 | 329 | 259 | 257 | 331 |
| **Hungary (n=988)** | | | | | | | | | |
|  | Gender | | Age (years) | | | Education levels | | Environmental attitudes | |
| Price | Male | Female | 18–34 | 35–54 | ≥55 | Below university | University | Ecocentric | Anthropocentric |
| 0.86 | 587 | 524 | 271 | 514 | 326 | 664 | 447 | 616 | 495 |
| 0.96 | 573 | 504 | 248 | 508 | 321 | 648 | 429 | 589 | 488 |
| 1.06 | 518 | 471 | 238 | 458 | 293 | 590 | 399 | 547 | 442 |
| 1.16 | 289 | 273 | 134 | 261 | 167 | 353 | 209 | 291 | 271 |
| 1.26 | 235 | 257 | 131 | 220 | 141 | 343 | 149 | 237 | 255 |
| **Croatia (n=506)** | | | | | | | | | |
|  | Gender | | Age (years) | | | Education levels | | Environmental attitudes | |
| Price | Male | Female | 18–34 | 35–54 | ≥55 | Below university | University | Ecocentric | Anthropocentric |
| 0.86 | 319 | 257 | 290 | 175 | 111 | 353 | 223 | 259 | 317 |
| 1.07 | 304 | 221 | 279 | 153 | 93 | 309 | 216 | 221 | 304 |
| 1.29 | 283 | 201 | 267 | 128 | 89 | 279 | 205 | 205 | 279 |
| 1.50 | 212 | 150 | 207 | 94 | 61 | 204 | 158 | 127 | 235 |
| 1.72 | 210 | 141 | 224 | 80 | 47 | 196 | 155 | 110 | 241 |
| **Belgium (n=950)** | | | | | | | | | |
|  | Gender | | Age (years) | | | Education levels | | Environmental attitudes | |
| Price | Male | Female | 18–34 | 35–54 | ≥55 | Below university | University | Ecocentric | Anthropocentric |
| 1.35 | 533 | 400 | 365 | 352 | 216 | 422 | 511 | 414 | 519 |
| 1.45 | 520 | 399 | 350 | 359 | 210 | 425 | 494 | 407 | 512 |
| 1.55 | 487 | 380 | 341 | 333 | 193 | 393 | 474 | 384 | 483 |
| 1.65 | 373 | 325 | 288 | 269 | 141 | 328 | 370 | 307 | 391 |
| 1.75 | 330 | 296 | 288 | 220 | 118 | 304 | 322 | 262 | 364 |

Note: All prices are in Euros.

## **Supplementary Table 11** Summary statistics of the demand for quantities of circular sliced pork in six EU countries

| Circular sliced pork | | | | | | | | | | | | | |
| --- | --- | --- | --- | --- | --- | --- | --- | --- | --- | --- | --- | --- | --- |
| Spain (n=1050) | | | |  | Poland (n=1040) | | | |  | Italy (n=755) | | | |
| Price  (€) | Median (unit) | Mean  (unit) | SD (unit) |  | Price  (€) | Median (unit) | Mean  (unit) | SD (unit) |  | Price  (€) | Median (unit) | Mean  (unit) | SD (unit) |
| 2.50 | 1 | 1.77 | 1.76 |  | 1.97 | 1 | 1.78 | 1.78 |  | 2.80 | 1 | 1.57 | 1.60 |
| 3.50 | 1 | 1.71 | 1.75 |  | 2.41 | 1 | 1.72 | 1.76 |  | 3.40 | 1 | 1.50 | 1.44 |
| 4.50 | 1 | 1.59 | 1.74 |  | 2.85 | 1 | 1.57 | 1.71 |  | 4.20 | 1 | 1.43 | 1.48 |
| 5.50 | 1 | 1.28 | 1.60 |  | 3.29 | 1 | 1.35 | 1.70 |  | 5.00 | 1 | 1.22 | 1.33 |
| **6.50** | **1** | **1.13** | **1.62** |  | **3.73** | **1** | **1.17** | **1.72** |  | **5.80** | **1** | **1.08** | **1.29** |
| Hungary (n=988) | | | |  | Croatia (n=506) | | | |  | Belgium (n=950) | | | |
| Price  (€) | Median (unit) | Mean  (unit) | SD (unit) |  | Price  (€) | Median (unit) | Mean  (unit) | SD (unit) |  | Price  (€) | Median (unit) | Mean  (unit) | SD (unit) |
| 2.93 | 1 | 1.87 | 2.25 |  | 3.09 | 2 | 1.93 | 1.96 |  | 4.50 | 1 | 1.40 | 1.64 |
| 3.13 | 1 | 1.84 | 2.18 |  | 3.55 | 2 | 1.88 | 1.88 |  | 5.50 | 1 | 1.36 | 1.58 |
| 3.32 | 1 | 1.82 | 2.13 |  | 4.02 | 1 | 1.64 | 1.80 |  | 6.50 | 1 | 1.29 | 1.52 |
| 3.52 | 1 | 1.49 | 1.95 |  | 4.48 | 1 | 1.32 | 1.58 |  | **7.50** | **1** | **1.04** | **1.35** |
| **3.72** | **1** | **1.25** | **1.89** |  | **4.95** | **1** | **1.15** | **1.61** |  | 8.50 | 0 | 0.93 | 1.41 |

## **Supplementary Table 12 Summary statistics of the demand for quantities of circular milk in six EU countries**

| Circular milk | | | | | | | | | | | | | |
| --- | --- | --- | --- | --- | --- | --- | --- | --- | --- | --- | --- | --- | --- |
| Spain (n=1050) | | | |  | Poland (n=1040) | | | |  | Italy (n=755) | | | |
| Price  (€) | Median (liter) | Mean  (liter) | SD (liter) |  | Price  (€) | Median (liter) | Mean  (liter) | SD (liter) |  | Price  (€) | Median (liter) | Mean  (liter) | SD (liter) |
| 0.55 | 2 | 3.02 | 4.03 |  | 0.43 | 2 | 2.49 | 3.01 |  | 0.85 | 1 | 2.08 | 2.72 |
| 0.65 | 2 | 2.85 | 3.81 |  | 0.47 | 1 | 2.28 | 2.74 |  | 0.95 | 1 | 1.98 | 2.59 |
| 0.75 | 1 | 2.66 | 3.79 |  | 0.54 | 1 | 2.13 | 2.66 |  | 1.05 | 1 | 1.79 | 2.22 |
| 0.85 | 1 | 2.09 | 3.30 |  | 0.60 | 1 | 1.70 | 2.37 |  | 1.15 | 1 | 1.46 | 1.99 |
| **0.95** | **1** | **1.79** | **2.96** |  | **0.67** | **1** | **1.51** | **2.31** |  | **1.25** | **1** | **1.27** | **1.88** |
| Hungary (n=988) | | | |  | Croatia (n=506) | | | |  | Belgium (n=950) | | | |
| Price  (€) | Median (liter) | Mean  (liter) | SD (liter) |  | Price  (€) | Median (liter) | Mean  (liter) | SD (liter) |  | Price  (€) | Median (liter) | Mean  (liter) | SD (liter) |
| 0.66 | 1 | 2.91 | 4.45 |  | 0.42 | 2 | 3.72 | 4.76 |  | 0.75 | 1 | 2.44 | 3.57 |
| 0.76 | 1 | 2.79 | 4.31 |  | 0.58 | 2 | 3.31 | 4.23 |  | 0.85 | 1 | 2.33 | 3.44 |
| **0.86** | **1** | **2.28** | **3.71** |  | 0.74 | 1 | 2.63 | 3.58 |  | 0.95 | 1 | 2.21 | 3.37 |
| 0.96 | 0 | 1.37 | 2.76 |  | **0.90** | **1** | **1.76** | **2.64** |  | **1.05** | **1** | **1.73** | **3.19** |
| 1.06 | 0 | 1.10 | 2.45 |  | 1.06 | 0 | 1.48 | 2.73 |  | 1.15 | 0 | 1.44 | 2.71 |

## **Supplementary Table 13** **Summary statistics of the demand for quantities of circular bread in six EU countries**

| Circular bread | | | | | | | | | | | | | |
| --- | --- | --- | --- | --- | --- | --- | --- | --- | --- | --- | --- | --- | --- |
| Spain (n=1050) | | | |  | Poland (n=1040) | | | |  | Italy (n=755) | | | |
| Price  (€) | Median (pack) | Mean  (pack) | SD (pack) |  | Price  (€) | Median (pack) | Mean  (pack) | SD (pack) |  | Price  (€) | Median (pack) | Mean  (pack) | SD (pack) |
| 0.70 | 1 | 1.74 | 1.84 |  | 0.71 | 1 | 1.80 | 1.86 |  | 0.75 | 1 | 1.61 | 1.57 |
| 0.90 | 1 | 1.73 | 1.85 |  | 1.04 | 1 | 1.62 | 1.75 |  | 0.85 | 1 | 1.57 | 1.52 |
| 1.10 | 1 | 1.51 | 1.67 |  | 1.37 | 1 | 1.33 | 1.65 |  | 0.95 | 1 | 1.48 | 1.45 |
| 1.30 | 1 | 1.30 | 1.73 |  | **1.70** | **1** | **1.06** | **1.60** |  | 1.05 | 1 | 1.20 | 1.37 |
| **1.50** | **1** | **1.19** | **1.69** |  | 2.03 | 0 | 0.95 | 1.67 |  | **1.15** | **1** | **1.14** | **1.39** |
| Hungary (n=988) | | | |  | Croatia (n=506) | | | |  | Belgium (n=950) | | | |
| Price  (€) | Median (pack) | Mean  (pack) | SD (pack) |  | Price  (€) | Median (pack) | Mean  (pack) | SD (pack) |  | Price  (€) | Median (pack) | Mean  (pack) | SD (pack) |
| 0.86 | 1 | 1.58 | 2.04 |  | 0.86 | 1 | 1.83 | 2.02 |  | 1.35 | 1 | 1.29 | 1.69 |
| 0.96 | 1 | 1.53 | 2.03 |  | 1.07 | 1 | 1.67 | 1.92 |  | 1.45 | 1 | 1.27 | 1.60 |
| **1.06** | **1** | **1.41** | **1.88** |  | **1.29** | **1** | **1.54** | **1.94** |  | 1.55 | 1 | 1.20 | 1.58 |
| 1.16 | 0 | 0.80 | 1.48 |  | 1.50 | 0 | 1.15 | 1.77 |  | **1.65** | **1** | **0.96** | **1.47** |
| 1.26 | 0 | 0.70 | 1.35 |  | 1.72 | 0 | 1.12 | 1.95 |  | 1.75 | 0 | 0.86 | 1.46 |

## **Supplementary Table 14** **NB regression of circular products in Spain**

|  | **Circular pork quantity** | **Circular milk quantity** | **Circular bread quantity** |
| --- | --- | --- | --- |
| **Variables** | **Coefficient (IRR^a^)** | **Coefficient (IRR)** | **Coefficient (IRR)** |
| **Gender** (vs male) |  |  |  |
| Female | -0.125*** (0.883) | -0.119*** (0.888) | -0.162*** (0.851) |
| **Age** (vs 18–24 years) |  |  |  |
| 45–54 years | -0.574*** (0.563) | -0.310*** (0.734) | -0.713*** (0.490) |
| >55 years | -0.669*** (0.512) | -0.359*** (0.699) | -0.812*** (0.444) |
| **Employment** (vs student) |  |  |  |
| Business owner | 0.183** (1.201) | 0.190 (1.209) | 0.128 (1.136) |
| On sick leave | 0.277 (1.320) | 0.117 (1.124) | 0.875** (2.400) |
| **Education** (vs university studies) |  |  |  |
| Elementary studies | -0.118* (0.889) | -0.119 (0.888) | 0.073 (1.076) |
| Secondary studies | -0.032 (0.968) | -0.127*** (0.881) | -0.135*** (0.873) |
| **Income covers expenditure** (vs always) |  |  |  |
| Sometimes | 0.083 (1.086) | -0.244* (0.783) | -0.291** (0.747) |
| **Stated finance**^b^ | 0.001 (1.000) | 0.021** (1.021) | 0.027*** (1.027) |
| **Environmental attitude**^c^ |  |  |  |
| Ecocentric consumers | 0.051*** (1.052) | 0.162*** (1.176) | 0.032* (1.033) |
| Anthropocentric consumers | 0.002 (1.002) | -0.103*** (0.902) | 0.027 (1.028) |
| **Price** |  |  |  |
| Circular pork price | -0.121*** (0.886) |  |  |
| Circular milk price |  | -1.383*** (0.251) |  |
| Circular bread price |  |  | -0.563*** (0.569) |
| _cons | 1.215*** | 1.910*** | 1.310*** |
| alpha^d^ | 0.400 | 1.195 | 0.420 |
| *p*-value | <0.001 | <0.001 | <0.001 |
| *N* | 4980 | 4595 | 4250 |

^a^ IRR: incidence-rate ratios.

^b^ Stated financial situation was measured using a 10-point Likert scale (1=very difficult, 10=very good).

^c^ The reduced New Ecological Paradigm (NEP) scale was used to measure this variable. Principal Component Analysis (PCA) was adopted. Ecocentric consumers and anthropocentric consumers were extracted.

^d^ Alpha was the estimated value of the dispersion parameter. If the dispersion parameter is equal to zero, the model reduces to the simpler Poisson model. If the dispersion parameter α is significantly greater than zero, the data are over-dispersed, and it is better to use a Negative Binomial (NB) model than a Poisson model. All α values were greater than zero. Therefore, the NB model fitted the data better than the Poisson model.

All categories were included in the regression models, but only statistically significant categories are reported in the table for clarity and due to word limitations.

* p<0.1, ** p<0.05, *** p<0.01

## **Supplementary Table 15 NB regression of circular products in Poland**

|  | **Circular pork quantity** | **Circular milk quantity** | **Circular bread quantity** |
| --- | --- | --- | --- |
| **Variables** | **Coefficient (IRR)** | **Coefficient (IRR)** | **Coefficient (IRR)** |
| **Gender** (vs male) |  |  |  |
| Female | -0.219*** (0.804) | -0.281*** (0.755) | -0.175*** (0.839) |
| **Age** (vs 18–24 years) |  |  |  |
| 45–54 years | -0.039 (0.962) | -0.227*** (0.797) | -0.396*** (0.673) |
| >55 years | -0.171*** (0.842) | -0.465*** (0.628) | -0.224*** (0.799) |
| **Employment** (vs student) |  |  |  |
| Business owner | 0.100 (1.105) | 0.420*** (1.522) | 0.361*** (1.435) |
| Retired | -0.632*** (0.531) | -0.233** (0.792) | -0.193* (0.824) |
| **Education** (vs university studies) |  |  |  |
| Secondary studies | -0.018 (0.982) | -0.008 (0.992) | 0.042 (1.043) |
| **Income covers expenditure** (vs always) |  |  |  |
| Never | -0.070 (0.932) | 0.044 (1.045) | -0.311*** (0.733) |
| **Stated finance** | 0.024*** (1.025) | 0.023** (1.023) | 0.044*** (1.045) |
| **Environmental attitude** |  |  |  |
| Ecocentric consumers | -0.033* (0.967) | -0.003 (0.997) | -0.167*** (0.847) |
| Anthropocentric consumers | 0.091*** (1.095) | 0.034** (1.035) | 0.090*** (1.094) |
| **Price** |  |  |  |
| Circular pork price | -0.057*** (0.945) |  |  |
| Circular milk price |  | -0.489*** (0.613) |  |
| Circular bread price |  |  | -0.122*** (0.885) |
| _cons | 1.261*** | 2.096*** | 1.033*** |
| alpha | 0.493 | 0.769 | 0.582 |
| *p*-value | <0.001 | <0.001 | <0.001 |
| *N* | 4600 | 4750 | 4410 |

* p<0.1, ** p<0.05, *** p<0.01

## **Supplementary Table 16 NB regression of circular products in Italy**

|  | **Circular pork quantity** | **Circular milk quantity** | **Circular bread quantity** |
| --- | --- | --- | --- |
| **Variables** | **Coefficient (IRR)** | **Coefficient (IRR)** | **Coefficient (IRR)** |
| **Gender** (vs male) |  |  |  |
| Female | -0.143*** (0.867) | -0.315*** (0.729) | -0.161*** (0.851) |
| **Age** (vs 18–24 years) |  |  |  |
| 45–54 years | -0.149** (0.862) | -0.289*** (0.749) | -0.046 (0.955) |
| >55 years | -0.477*** (0.621) | -0.253*** (0.776) | -0.298*** (0.743) |
| **Employment** (vs student) |  |  |  |
| Retired | -0.182* (0.834) | -0.289** (0.749) | -0.509*** (0.601) |
| **Education** (vs university studies) |  |  |  |
| Elementary studies | -0.128 (0.880) | -0.385*** (0.680) | -0.139 (0.870) |
| Secondary studies | 0.015 (1.016) | -0.186*** (0.831) | 0.013 (1.013) |
| **Income covers expenditure** (vs always) |  |  |  |
| Rarely | -0.025 (0.975) | -0.143*** (0.867) | -0.037 (0.964) |
| Never | -0.494*** (0.610) | -0.399*** (0.671) | -0.025 (0.975) |
| **Stated finance** | -0.009 (0.991) | -0.008 (0.992) | <0.001 (1.000) |
| **Environmental attitude** |  |  |  |
| Ecocentric consumers | -0.005 (0.995) | -0.020 (0.980) | -0.041** (0.960) |
| Anthropocentric consumers | 0.040** (1.040) | 0.022 (1.023) | 0.007 (1.007) |
| **Price** |  |  |  |
| Circular pork price | -0.126*** (0.882) |  |  |
| Circular milk price |  | -1.273*** (0.280) |  |
| Circular bread price |  |  | -0.953*** (0.385) |
| _cons | 1.402*** | 2.560*** | 1.631*** |
| alpha | 0.212 | 0.692 | 0.213 |
| *p*-value | <0.001 | <0.001 | <0.001 |
| *N* | 3285 | 2960 | 2565 |

* p<0.1, ** p<0.05, *** p<0.01

## **Supplementary Table 17 NB regression of circular products in Hungary**

|  | **Circular pork quantity** | **Circular milk quantity** | **Circular bread quantity** |
| --- | --- | --- | --- |
| **Variables** | **Coefficient (IRR)** | **Coefficient (IRR)** | **Coefficient (IRR)** |
| **Gender** (vs male) |  |  |  |
| Female | -0.147*** (0.863) | -0.081 (0.922) | -0.117** (0.889) |
| **Age** (vs 18–24 years) |  |  |  |
| 45–54 years | -0.253*** (0.777) | -0.092 (0.912) | 0.012 (1.012) |
| >55 years | -0.293*** (0.746) | -0.290** (0.748) | -0.049 (0.952) |
| **Employment** (vs student) |  |  |  |
| Business owner | 0.251** (1.285) | -0.031 (0.970) | 0.445*** (1.560) |
| Employee (full time) | 0.284*** (1.328) | 0.317** (1.372) | 0.251* (1.286) |
| **Education** (vs university studies) |  |  |  |
| Elementary studies | -0.372*** (0.690) | -0.304*** (0.738) | -0.417*** (0.659) |
| Secondary studies | -0.201*** (0.818) | -0.237*** (0.789) | -0.095* (0.909) |
| **Income covers expenditure** (vs always) |  |  |  |
| Rarely | 0.077 (1.080) | -0.202*** (0.817) | 0.036 (1.037) |
| Never | 0.018 (1.018) | -0.545*** (0.580) | -0.291*** (0.747) |
| **Stated finance** | 0.014 (1.015) | 0.019 (1.019) | 0.033*** (1.033) |
| **Environmental attitude** |  |  |  |
| Ecocentric consumers | 0.116*** (1.123) | 0.071*** (1.073) | 0.045* (1.046) |
| Anthropocentric consumers | -0.016 (0.984) | -0.043* (0.957) | -0.066*** (0.936) |
| **Price** |  |  |  |
| Circular pork price | -0.520*** (0.594) |  |  |
| Circular milk price |  | -2.630*** (0.072) |  |
| Circular bread price |  |  | -2.230*** (0.108) |
| _cons | 2.274*** | 3.008*** | 2.269*** |
| alpha | 0.638 | 1.412 | 0.760 |
| *p*-value | <0.001 | <0.001 | <0.001 |
| *N* | 3965 | 3730 | 3270 |

* p<0.1, ** p<0.05, *** p<0.01

## **Supplementary Table 18 NB regression of circular products in Croatia**

|  | **Circular pork quantity** | **Circular milk quantity** | **Circular bread quantity** |
| --- | --- | --- | --- |
| **Variables** | **Coefficient (IRR)** | **Coefficient (IRR)** | **Coefficient (IRR)** |
| **Gender** (vs male) |  |  |  |
| Female | -0.063 (0.939) | -0.011 (0.989) | -0.025 (0.975) |
| **Age** (vs 18–24 years) |  |  |  |
| 45–54 years | -0.326*** (0.722) | -0.214** (0.807) | -0.568*** (0.567) |
| >55 years | -0.491*** (0.612) | -0.341*** (0.711) | -0.453*** (0.636) |
| **Employment** (vs student) |  |  |  |
| Business owner | 0.375*** (1.455) | 0.158 (1.171) | 0.255* (1.291) |
| Employee (full time) | 0.223** (1.250) | 0.280** (1.323) | 0.275** (1.317) |
| **Education** (vs university studies) |  |  |  |
| Elementary studies | -0.473** (0.623) | -0.821*** (0.440) | -0.268 (0.765) |
| **Income covers expenditure** (vs always) |  |  |  |
| Rarely | -0.075 (0.928) | -0.255*** (0.775) | 0.012 (1.013) |
| Never | -0.168** (0.846) | -0.116 (0.891) | 0.121 (1.129) |
| **Stated finance** | 0.024** (1.024) | 0.008 (1.008) | 0.011 (1.011) |
| **Environmental attitude** |  |  |  |
| Ecocentric consumers | -0.066*** (0.936) | -0.137*** (0.872) | -0.180*** (0.835) |
| Anthropocentric consumers | 0.057** (1.058) | 0.080*** (1.083) | 0.042 (1.043) |
| **Price** |  |  |  |
| Circular pork price | -0.309*** (0.734) |  |  |
| Circular milk price |  | -1.741*** (0.175) |  |
| Circular bread price |  |  | -0.718*** (0.488) |
| _cons | 1.657*** | 2.265*** | 1.419*** |
| alpha | 0.506 | 1.357 | 0.709 |
| *p*-value | <0.001 | <0.001 | <0.001 |
| *N* | 2280 | 2355 | 1570 |

* p<0.1, ** p<0.05, *** p<0.01

## **Supplementary Table 19 NB regression of circular products in Belgium**

|  | **Circular pork quantity** | **Circular milk quantity** | **Circular bread quantity** |
| --- | --- | --- | --- |
| **Variables** | **Coefficient (IRR)** | **Coefficient (IRR)** | **Coefficient (IRR)** |
| **Gender** (vs male) |  |  |  |
| Female | -0.213*** (0.808) | -0.347*** (0.707) | -0.183*** (0.832) |
| **Age** (vs 18–24 years) |  |  |  |
| 45–54 years | -0.550*** (0.577) | -0.560*** (0.571) | -0.708*** (0.493) |
| >55 years | -0.671*** (0.511) | -0.729*** (0.482) | -0.820*** (0.440) |
| **Employment** (vs student) |  |  |  |
| Business owner | 0.726*** (2.067) | 0.419*** (1.520) | 0.340*** (1.404) |
| Employee (full time) | 0.599*** (1.820) | 0.585*** (1.795) | 0.243** (1.276) |
| **Education** (vs university studies) |  |  |  |
| Elementary studies | 0.053 (1.055) | -0.399*** (0.671) | -0.120 (0.887) |
| Secondary studies | -0.070* (0.932) | -0.090* (0.914) | -0.198*** (0.820) |
| **Income covers expenditure** (vs always) |  |  |  |
| Rarely | 0.062 (1.064) | -0.205*** (0.815) | 0.068 (1.070) |
| Never | -0.281*** (0.755) | -0.311*** (0.733) | -0.085 (0.919) |
| **Stated finance** | -0.023** (0.977) | -0.058*** (0.944) | -0.002 (0.998) |
| **Environmental attitude** |  |  |  |
| Ecocentric consumers | 0.068*** (1.071) | 0.177*** (1.194) | 0.080*** (1.084) |
| Anthropocentric consumers | 0.003 (1.003) | -0.006 (0.994) | 0.012 (1.012) |
| **Price** |  |  |  |
| Circular pork price | -0.109*** (0.897) |  |  |
| Circular milk price |  | -1.409*** (0.244) |  |
| Circular bread price |  |  | -1.087*** (0.337) |
| _cons | 1.038*** | 2.587*** | 2.109*** |
| alpha | 0.418 | 1.494 | 0.570 |
| *p*-value | <0.001 | <0.001 | <0.001 |
| *N* | 3895 | 3850 | 3620 |

* p<0.1, ** p<0.05, *** p<0.01

## **Supplementary Table 20 Correlation between consumers’ environmental attitude, opinions about the impact of farming systems on the environment, and opinions about the environmental sustainability of dietary patterns**

|  | Conventional | Organic | Circular | Vegetarian | Vegan | Flexitarian | Non-restricted diets |
| --- | --- | --- | --- | --- | --- | --- | --- |
| **Spain** | | | | | | | |
| Ecocentric | -0.101** | 0.431** | 0.275** | 0.275** | 0.314** | 0.306** | -0.193** |
| Anthropocentric | 0.390** | 0.041 | 0.087** | 0.063* | 0.023 | 0.083** | 0.309** |
| **Poland** | | | | | | | |
| Ecocentric | -0.063* | 0.363** | 0.196** | 0.304** | 0.295** | 0.307** | -0.188** |
| Anthropocentric | 0.311** | -0.036 | 0.104** | -0.004 | -0.055 | -0.013 | 0.344** |
| **Italy** | | | | | | | |
| Ecocentric | -0.174** | 0.316** | -0.170** | 0.279** | 0.286** | 0.335** | -0.336** |
| Anthropocentric | 0.333** | 0.060 | 0.019 | 0.057 | -0.019 | 0.082* | 0.348** |
| **Hungary** | | | | | | | |
| Ecocentric | -0.088** | 0.329** | 0.344** | 0.188** | 0.212** | 0.245** | -0.110** |
| Anthropocentric | 0.220** | 0.146** | 0.037** | 0.115** | 0.072** | 0.126** | 0.196** |
| **Croatia** | | | | | | | |
| Ecocentric | -0.087** | 0.338** | 0.161** | 0.179** | 0.159** | 0.252** | -0.184** |
| Anthropocentric | 0.261** | -0.092** | 0.017 | 0.037 | -0.026 | -0.029 | 0.244** |
| **Belgium** | | | | | | | |
| Ecocentric | -0.198** | 0.325** | 0.281** | 0.309** | 0.323** | 0.281** | -0.241** |
| Anthropocentric | 0.301** | 0.048** | 0.033* | 0.030* | -0.037* | 0.078** | 0.387** |

* p<0.05, ** p<0.01

# Supplementary Figures


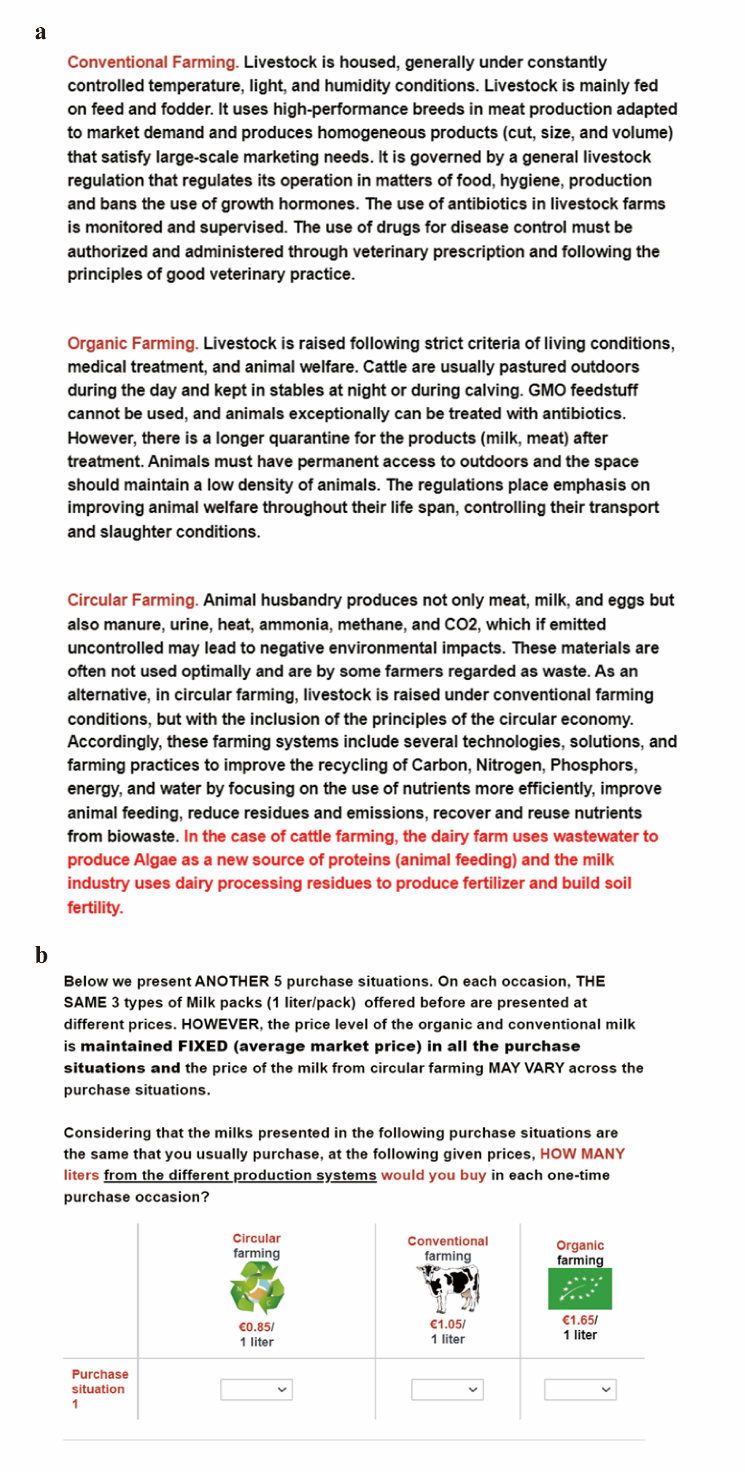


## **Supplementary Figure 1 An example** **of milk in the OECE and definitions of various farming systems for milk**. a, Definitions of various farming systems for milk that respondents were presented with before making choices in OECEs. b, An example of milk in the OECE.


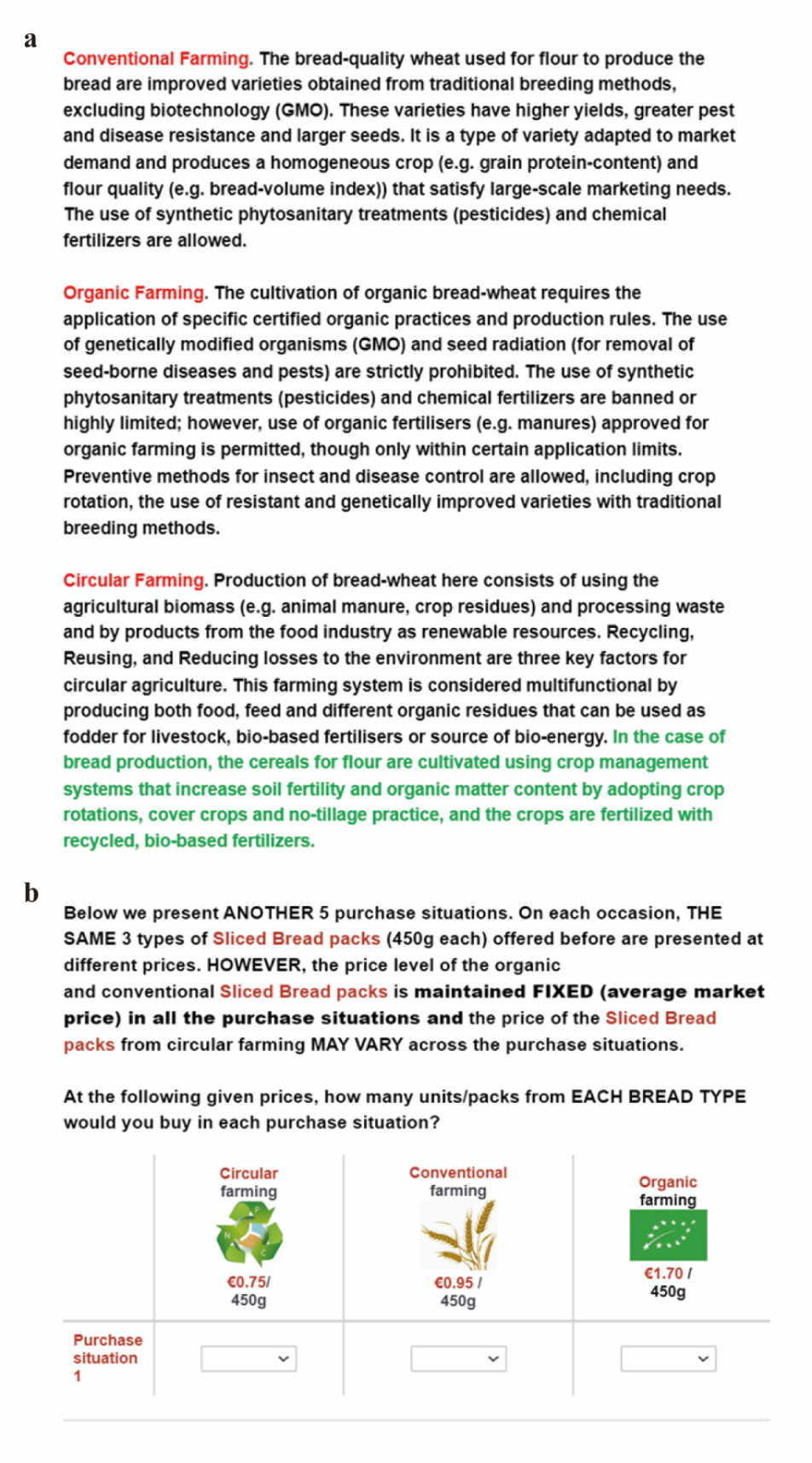


## **Supplementary Figure 2** **An example of sliced bread in the OECE and definitions of various farming systems for bread**. a, Definitions of various farming systems for bread that respondents were presented with before making choices in OECEs. b, An example of sliced bread in the OECE.


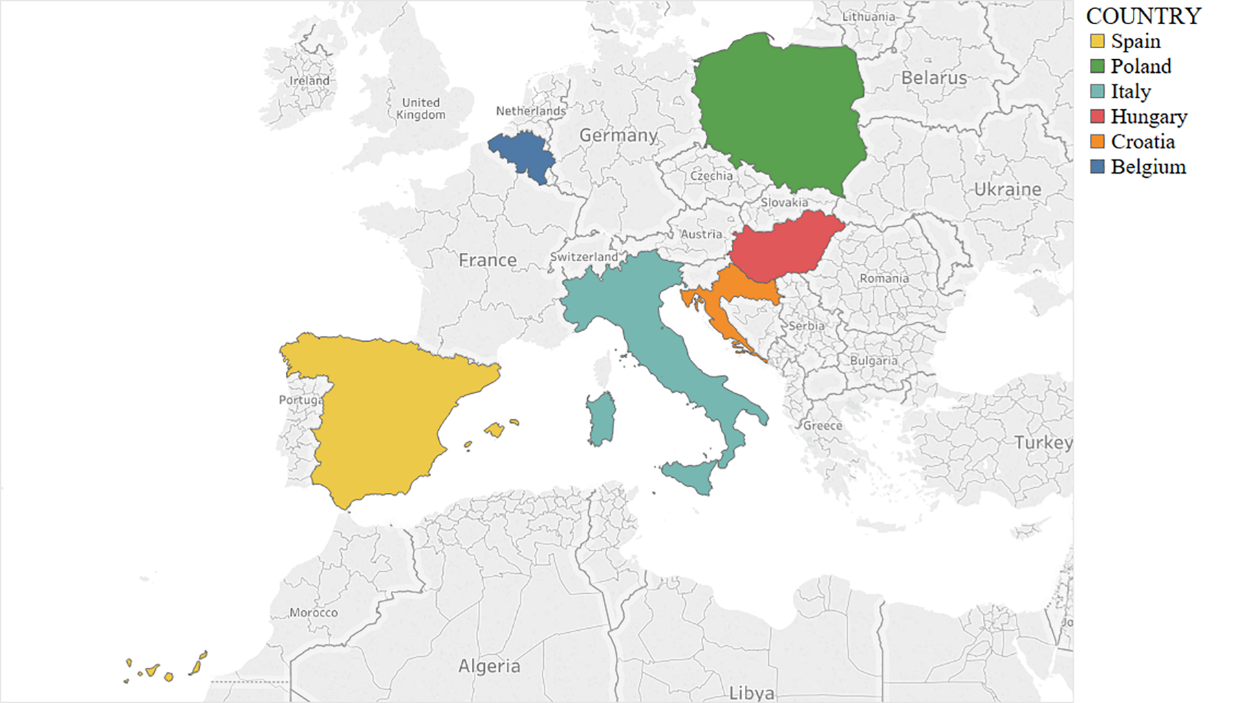


## **Supplementary Figure 3** **Geographical location of the study area of this research**


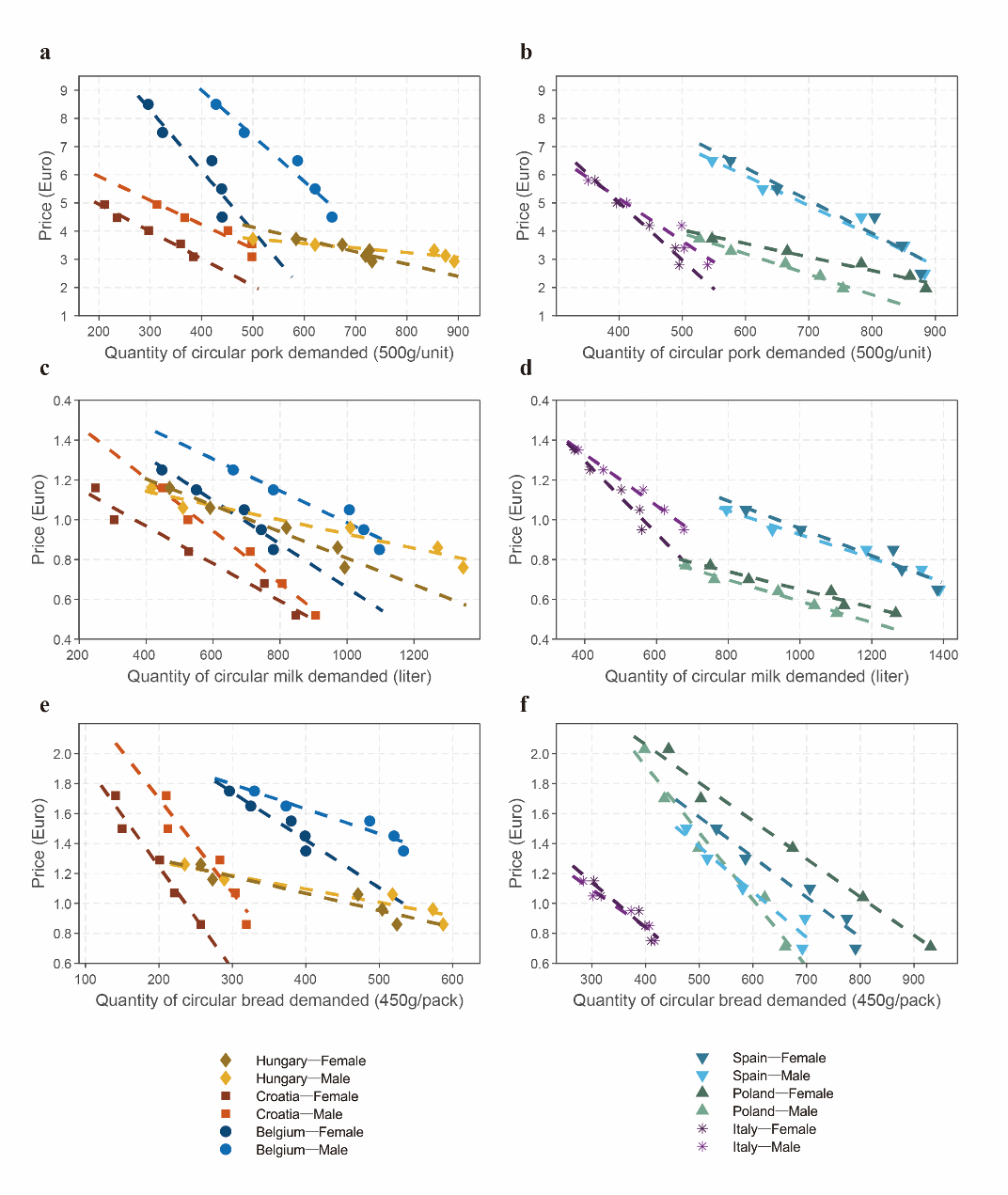


Supplementary Figure 4 Observed demand for circular food products by gender in six countries. **a, b,** Quantity of circular pork desired by gender in each country. **c, d,** Quantity of circular milk desired by gender in each country. **e, f,** Quantity of circular bread desired by gender in each country.


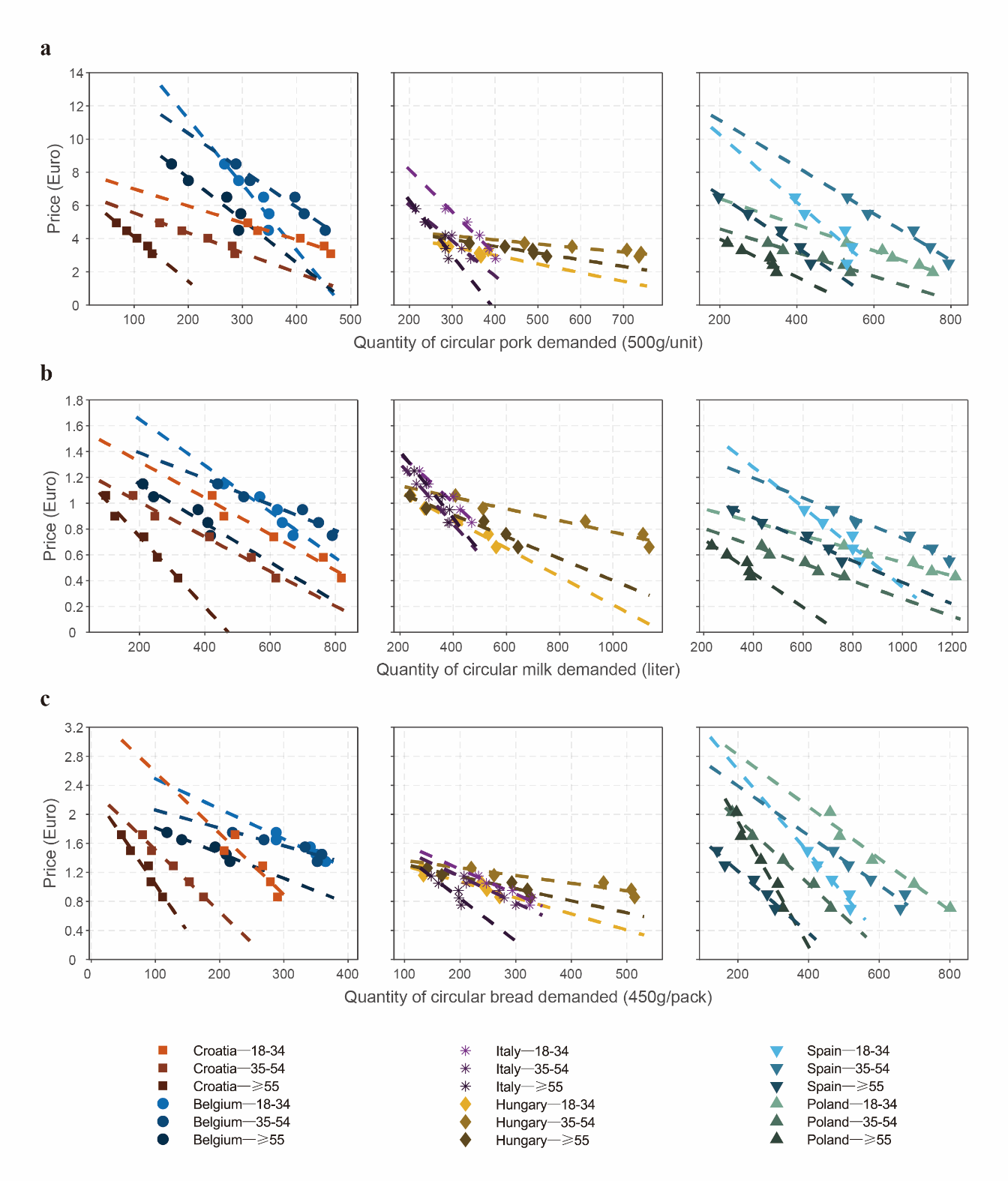


Supplementary Figure 5 Observed demand for circular food products by age in six countries. **a,** Quantity of circular pork desired by age in each country. **b,** Quantity of circular milk desired by age in each country. **c,** Quantity of circular bread desired by age in each country.


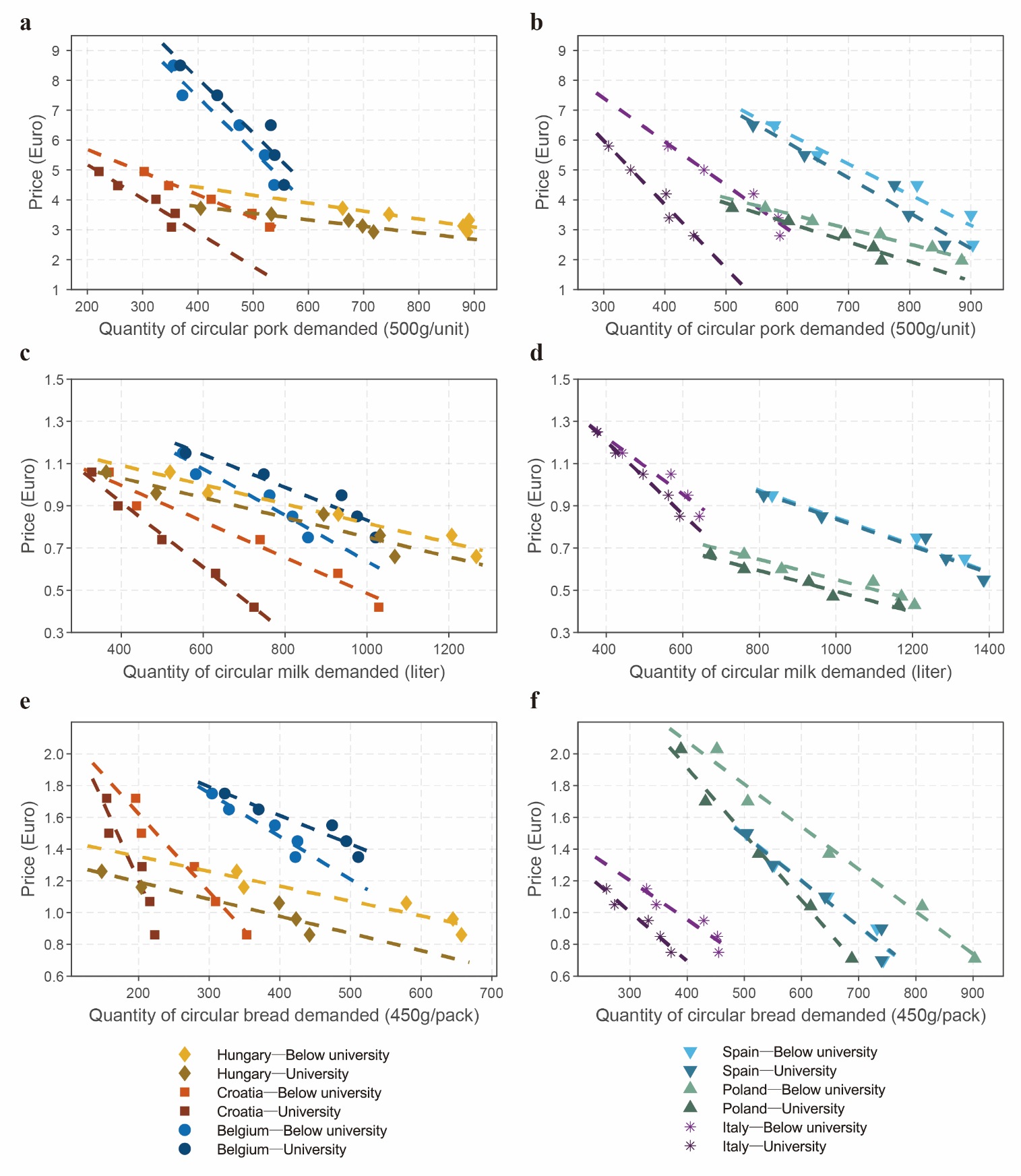


Supplementary Figure 6 Observed demand for circular food products by education levels in six countries. **a, b,** Quantity of circular pork desired by education levels in each country. **c, d,** Quantity of circular milk desired by education levels in each country. **e, f,** Quantity of circular bread desired by education levels in each country.


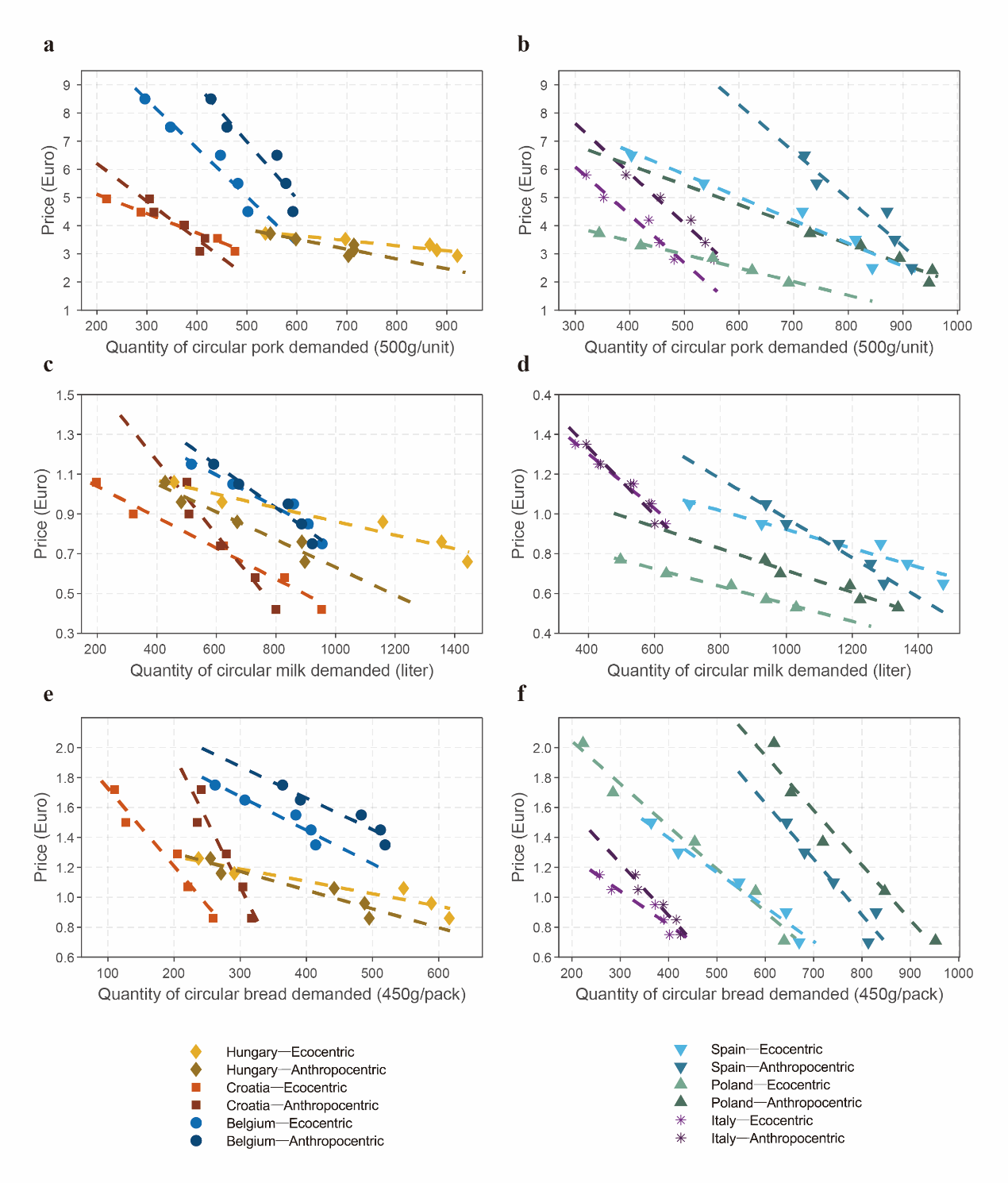


Supplementary Figure 7 Observed demand for circular food products by environmental attitudes in six countries. **a, b,** Quantity of circular pork desired by environmental attitudes in each country. **c, d,** Quantity of circular milk desired by environmental attitudes in each country. **e, f,** Quantity of circular bread desired by environmental attitudes in each country.

**
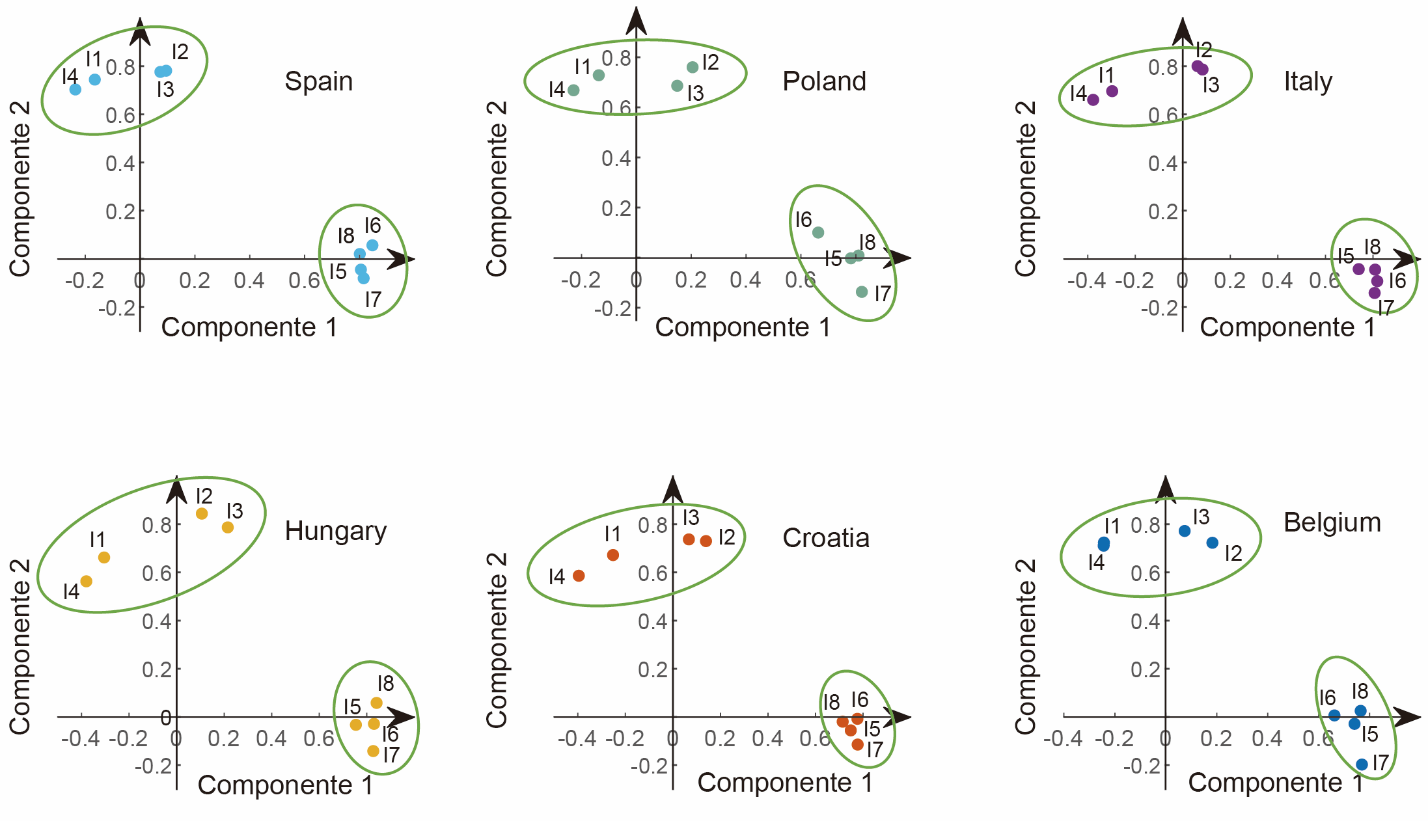
**

## **Supplementary Figure 8 PCA results of environmental attitudes.** I1–I8: Item 1 to Item 8.


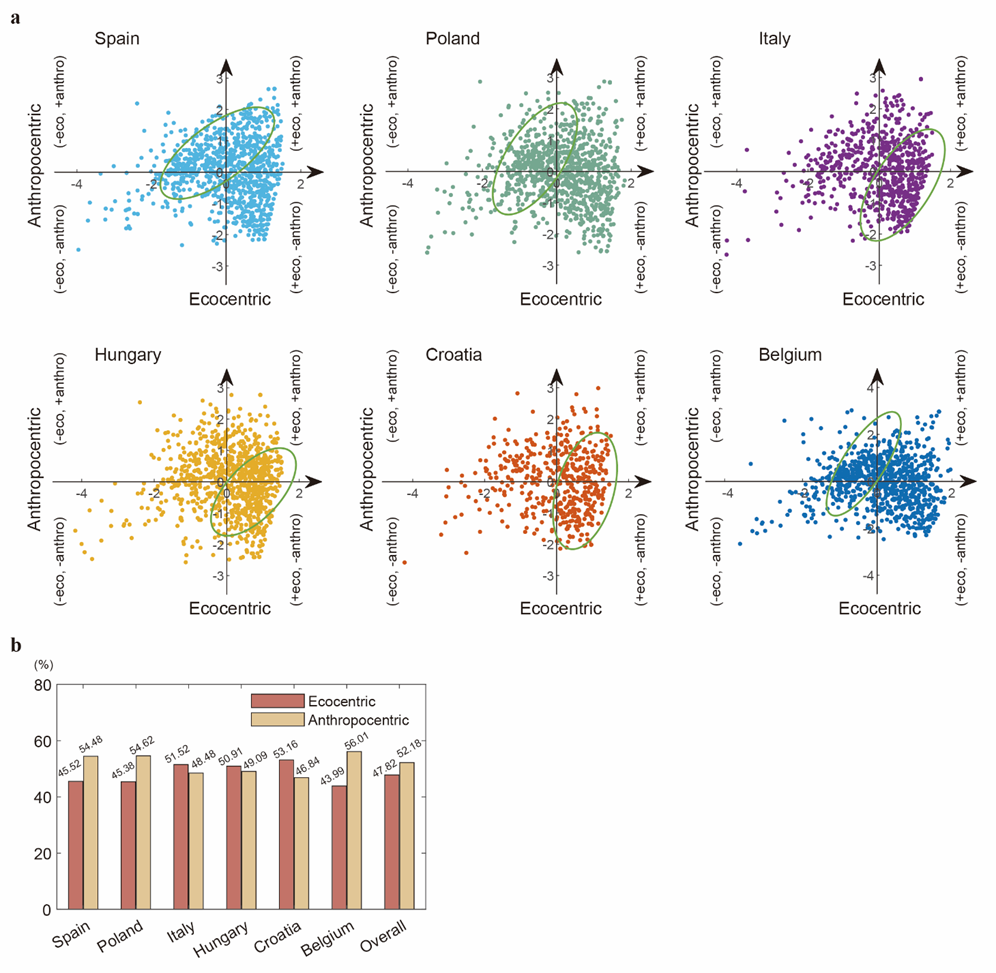


## **Supplementary Figure 9** **Consumers’ environmental attitudes in each country**. a, Distribution of consumers in ecocentric and anthropocentric dimensions in each country. b, Proportion of ecocentric and anthropocentric environmental attitudes in each country and overall.

# Full Questionnaire

*(Note: The questionnaire was conducted in the format of an online survey. Below is the* ***original version of the questionnaire we designed****, and the prices in this questionnaire are shown as an example of Italian prices.* ***In the actual online distribution of the questionnaire, respondents were presented with the three open-ended choice experiments in a randomized order. The order of the purchase situations and products within the cards presented to consumers was also randomized.*** *Nonetheless, a fixed order is presented here in this static version for clarity.)*

Q1.1  
Our project is working on innovative solutions at the farm level by applying the principles of the circular economy to agriculture. Accordingly, these farming systems include several technologies, solutions, and practices that improve the current cycle of carbon, nitrogen, phosphorus, energy, and water. It focuses on the more efficient use of nutrients to improve animal feeding, reduce residues and emissions, and recover and reuse nutrients from biowaste.

Survey Objective:

The objective of this survey is to analyze consumers’ demand and willingness to pay a premium for pork, milk, and bread products obtained from sustainable circular systems.

Duration: The questionnaire will take approximately 15 minutes to complete.

Conditions:

The results of this survey have the character of public research and all information collected will be treated CONFIDENTIALLY and strictly ANONYMOUSLY. Responses will NEVER be provided to third parties. Please note that if you CANNOT or DO NOT WANT to proceed, you MAY stop. Remember that you can exit the questionnaire without completing it and return to it at any time at the point where you left off. Your privacy and data protection in the survey are in line with the GDPR regulation (2016/679 EC), including the right to access your data, rectify them, erase them, restrict the processing, data portability, and object to processing.

Q1.1 Do you want to participate?

- I agree to participate
- I refuse to participate

**Section 1 Socio-demographic Information**

Q1.2 What is your gender?

- Male
- Female

Q1.3 What is your age?

- 18–24 years
- 25–34 years
- 35–44 years
- 45–54 years
- More than 55 years

Q1.4 How would you describe your current financial situation?

5

10

Very good

1

Very difficult

|  |
| --- |

Q1.5 Does the level of your monthly income cover your household expenditure?

- Always
- Very Often
- Sometimes
- Rarely
- Never

Q1.6 What is your education level?

- Not completed elementary studies
- Elementary studies
- Secondary studies
- University studies

Q1.7 What is your current employment status?

- Student
- Self-employed/Business owner (full time)
- Employee (full time)
- A homemaker
- On sick leave
- Unemployed
- Retired
- Unable to work

Q1.8 Who is mainly responsible for your household food shopping?

- Exclusively me
- Mostly me
- I and someone else
- Mostly someone else
- Exclusively someone else

**Section 2 Open-ended Choice Experiments in 3 Cases**

***Case 1: Pork Loin***

Q2.1 Do you eat pork?

- Yes
- No

Q2.2 Do you purchase pork?

- Yes
- No

***OPEN-ENDED CHOICE EXPERIMENT PORK LOIN***

**Below you are presented with definitions of different farming systems and the definition of pork from circular farming. The pork loins are obtained from different production systems: Conventional farming, Organic farming, and Circular farming.**

Conventional Farming. Livestock is housed, generally under constantly-controlled temperature, light, and humidity conditions. Livestock is mainly fed on feed and fodder. It uses high-performance breeds in meat production adapted to market demand and produces homogeneous products (cut, size, and volume) that satisfy large-scale marketing needs. It is governed by a general livestock regulation that regulates its operation in matters of food, hygiene, production and bans the use of growth hormones. The use of antibiotics in livestock farms is monitored and supervised. The use of drugs for disease control must be authorized and administered through veterinary prescription and must follow the principles of good veterinary practice.

Organic Farming. Livestock is raised following strict criteria of living conditions, medical treatment, and animal welfare. Livestock is fed with organic certificate fodder. GMO feedstuff cannot be used, and animals exceptionally can be treated with antibiotics. However, there is a longer quarantine for the products (meat) after treatment. Animals must have permanent access to outdoors, and the space should maintain a low density of animals. The regulations place emphasis on improving animal welfare throughout their life span, controlling their transport and slaughter conditions.

Circular Farming. Animal husbandry produces not only meat, milk, and eggs but also manure, urine, heat, ammonia, methane, and CO_2_, which, if emitted uncontrolled, may lead to negative environmental impacts. These materials are often not used optimally and are by some farmers regarded as waste. As an alternative, in circular farming, livestock is raised under conventional farming conditions, but with the inclusion of the principles of the circular economy. Accordingly, these farming systems include several technologies, solutions, and farming practices to improve the recycling of carbon, nitrogen, phosphorus, energy, and water by focusing on the use of nutrients more efficiently, improving animal feeding, reducing residues and emissions, and recovering and reusing nutrients from biowaste.  In the case of pig farming, pig slurry and manure are treated to produce bio-energy (biogas) and bio-based fertilizers using a combination of techniques.

Q2.3
Below we present 5 purchase situations. On each occasion, THE SAME 3 types of sliced pork loin packs (500 g each) are presented at different prices. HOWEVER, the price level of the organic and conventional loin remains **FIXED** (average market price) in all the purchase situations, while the price of the loin from circular farming MAY **VARY** across the purchase situations.

**At the following given prices, HOW MANY units/packs (500 g each) from EACH LOIN TYPE would you buy in each purchase situation? (from 0 to 10 units/packs)**


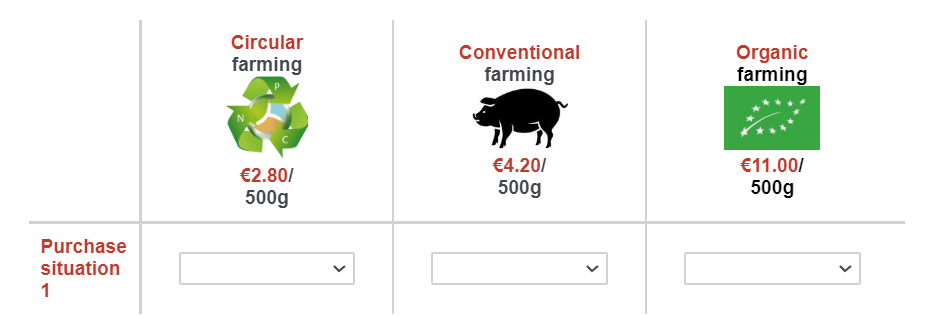


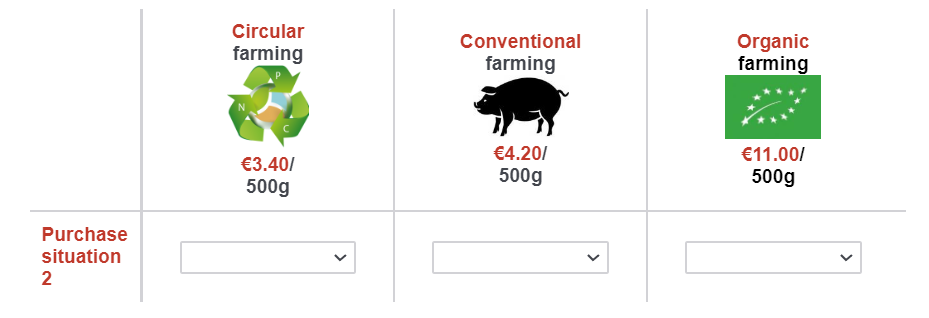


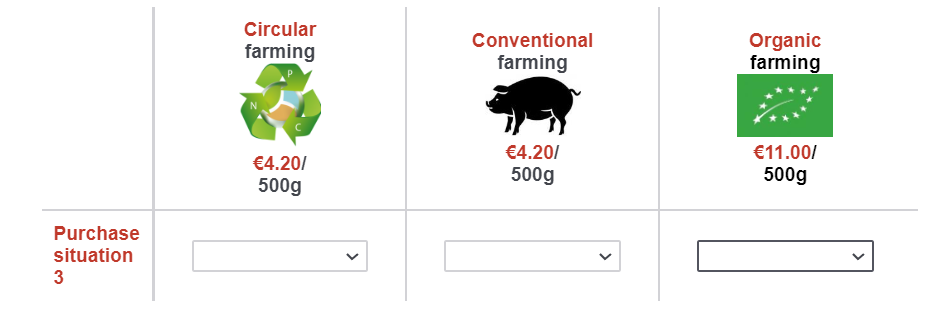


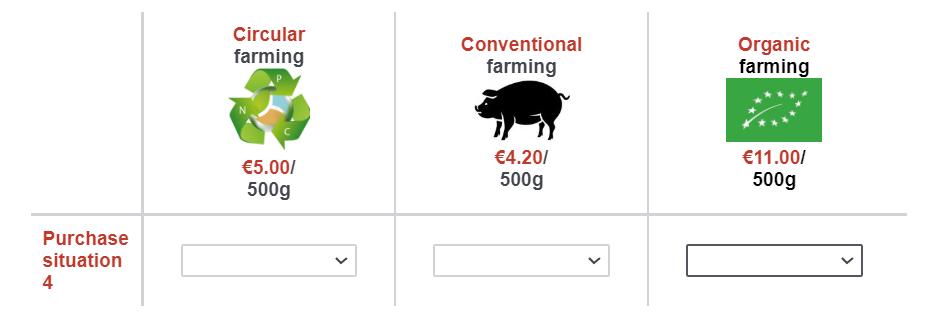


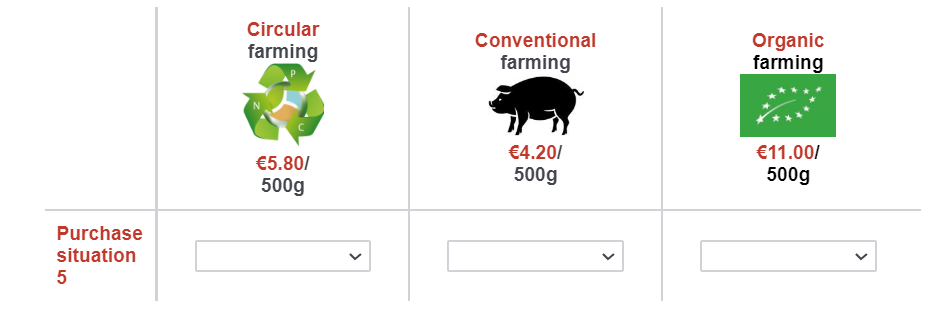


***Case 2: Milk***

Q2.4 Do you drink cows’ milk (any type)?

- Yes
- No

Q2.5 Do you purchase cows’ milk (any type)?

- Yes
- No

***OPEN-ENDED CHOICE EXPERIMENT MILK***

**Below you are presented with definitions of the different farming systems and the definition of milk from circular farming. The MILK is obtained from different production systems: Conventional farming, Organic farming, and Circular farming.**

Conventional Farming. Livestock is housed, generally under constantly-controlled temperature, light, and humidity conditions. Livestock is mainly fed on feed and fodder. It is governed by a general livestock regulation that regulates its operation in matters of food, hygiene, production and bans the use of growth hormones. The use of antibiotics in livestock farms is monitored and supervised. The use of drugs for disease control must be authorized and administered through veterinary prescription and must follow the principles of good veterinary practice.

Organic Farming. Livestock is raised following strict criteria of living conditions, medical treatment, and animal welfare. Cattle are usually pastured outdoors during the day and kept in stables at night or during calving. GMO feedstuff cannot be used, and animals exceptionally can be treated with antibiotics. However, there is a longer quarantine for the products (milk, meat) after treatment. Animals must have permanent access to outdoors and the space should maintain a low density of animals. The regulations place emphasis on improving animal welfare throughout their life span, controlling their transport and slaughter conditions.

Circular Farming. Animal husbandry produces not only meat, milk, and eggs but also manure, urine, heat, ammonia, methane, and CO_2_, which, if emitted uncontrolled, may lead to negative environmental impacts. These materials are often not used optimally and are by some farmers regarded as waste. As an alternative, in circular farming, livestock is raised under conventional farming conditions, but with the inclusion of the principles of the circular economy. Accordingly, these farming systems include several technologies, solutions, and farming practices to improve the recycling of carbon, nitrogen, phosphorus, energy, and water by focusing on the use of nutrients more efficiently, improving animal feeding, reducing residues and emissions, recovering and reusing nutrients from biowaste. In the case of cattle farming, the dairy farm uses wastewater to produce Algae as a new source of protein (animal feeding), and the milk industry uses dairy processing residues to produce fertilizer and build soil fertility.

Q2.6
Below we present 5 purchase situations. On each occasion, THE SAME 3 types of Milk packs (1 liter/pack) are presented at different prices. HOWEVER, the price level of the organic and conventional milk remains **FIXED** (average market price) in all the purchase situations, and the price of the milk from circular farming MAY **VARY** across the purchase situations.

**Considering that the milks presented in the following purchase situations are the same that you usually purchase, at the following given prices, HOW MANY liters (1 liter/pack) from the different production systems would you buy in each one-time purchase occasion? (from 0 to 24 liters/packs)**


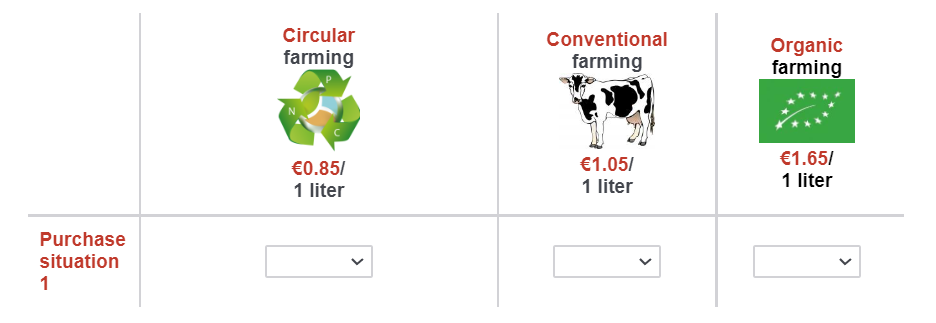


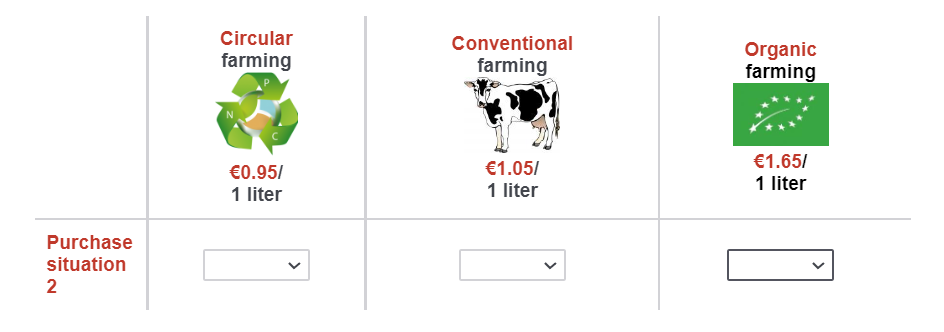


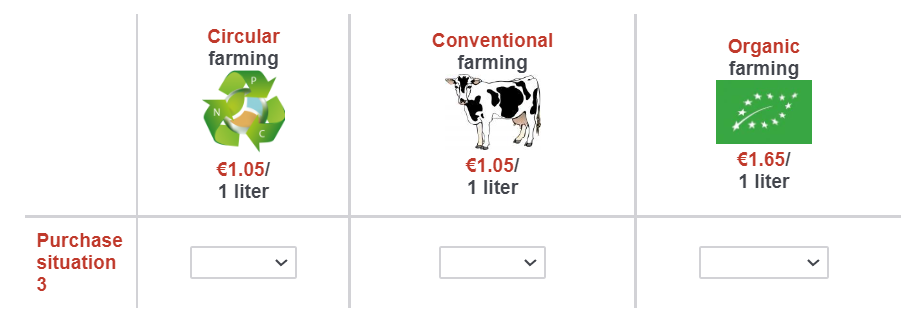


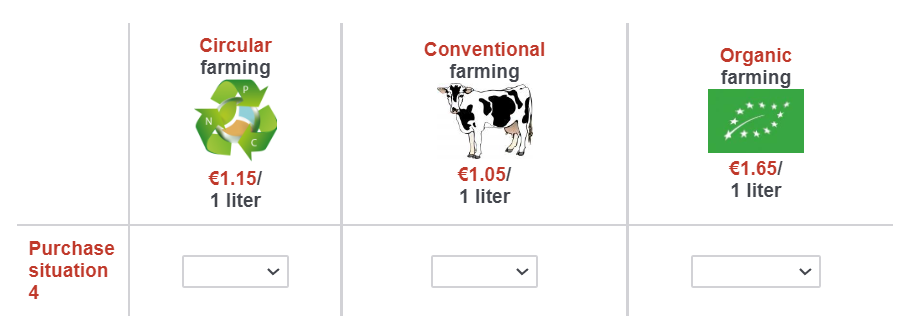


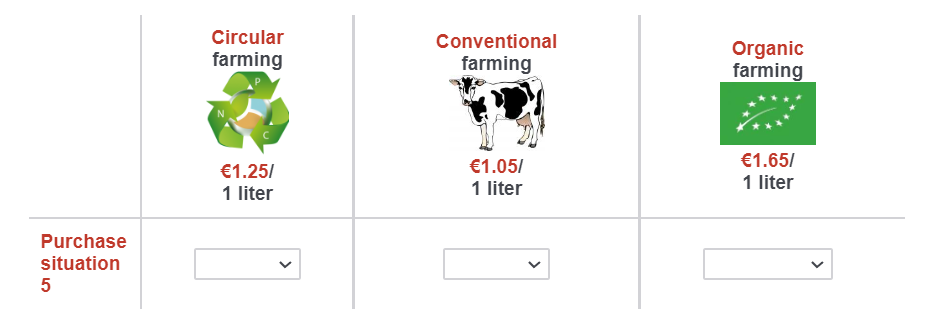


***Case 3: Sliced Bread***

Q2.7 Do you consume sliced bread?

- Yes
- No

Q2.8 Do you purchase sliced bread?

- Yes
- No

***OPEN-ENDED CHOICE EXPERIMENT SLICED BREAD***

**Below you are presented with definitions of the different farming systems and the definition of bread from circular farming. The SLICED BREAD packs are produced with wheat flour obtained from different production systems: Conventional farming, Organic farming, and Circular farming.**

Conventional Farming. The bread-quality wheat used for flour to produce the bread is improved varieties obtained from traditional breeding methods, excluding biotechnology (GMO). These varieties have higher yields, greater pest and disease resistance, and larger seeds. It is a type of variety adapted to market demand and produces a homogeneous crop (e.g., grain protein-content) and flour quality (e.g., bread-volume index) that satisfy large-scale marketing needs. The use of synthetic phytosanitary treatments (pesticides) and chemical fertilizers is allowed.

Organic Farming. The cultivation of organic bread-wheat requires the application of specific certified organic practices and production rules. The use of genetically-modified organisms (GMO) and seed radiation (for removal of seed-borne diseases and pests) is strictly prohibited. The use of synthetic phytosanitary treatments (pesticides) and chemical fertilizers is banned or severely limited; however, the use of organic fertilisers (e.g., manures) approved for organic farming is permitted, though only within certain application limits. Preventive methods for insect and disease control are allowed, including crop rotation, and the use of resistant and genetically-improved varieties with traditional breeding methods.

Circular Farming. Production of bread-wheat here consists of using the agricultural biomass (e.g., animal manure, crop residues) and processing waste and by-products of the food industry as renewable resources. Recycling, reusing, and reducing losses to the environment are three key factors for circular agriculture. This farming system is considered multifunctional by producing both food, feed and different organic residues that can be used as fodder for livestock, bio-based fertilisers or as a source of bio-energy. In the case of bread, cereals are cultivated using crop management systems that increase soil fertility and organic matter content by adopting crop rotations, cover crops and no-tillage practices, and the crops are fertilized with recycled, bio-based fertilizers.

Q2.9
Below we present 5 purchase situations. On each occasion, THE SAME 3 types of Sliced Bread packs (450 g each) are presented at different prices. HOWEVER, the price level of the organic and conventional Sliced Bread packs remains **FIXED** (average market price) in all the purchase situations and the price of the Sliced Bread packs from circular farming MAY **VARY** across the purchase situations.

**At the following given prices, HOW MANY units/packs (450 g each) from EACH BREAD TYPE would you buy in each purchase situation? (from 0 to 10 units/packs)**


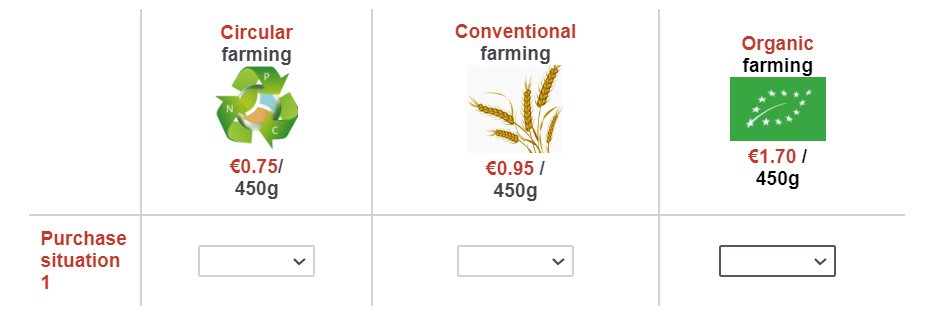


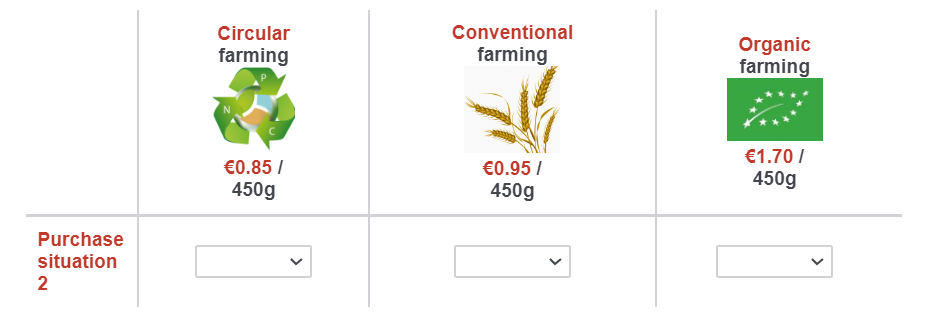


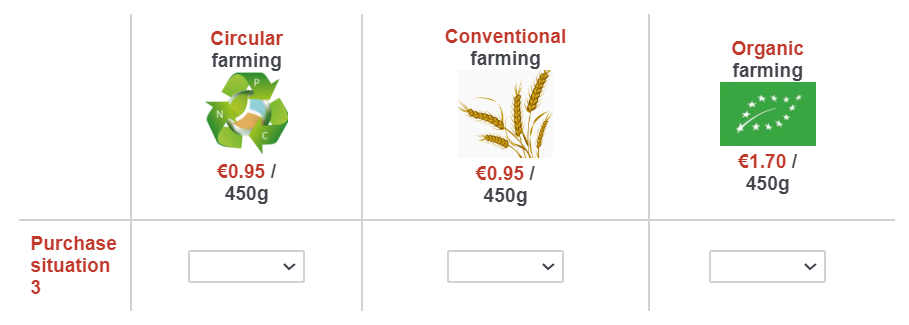


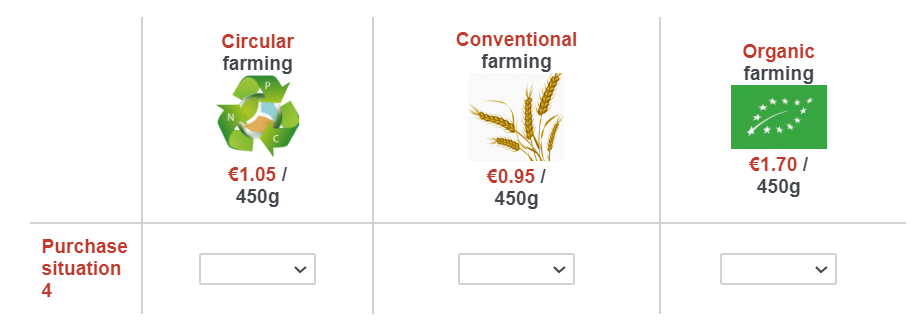


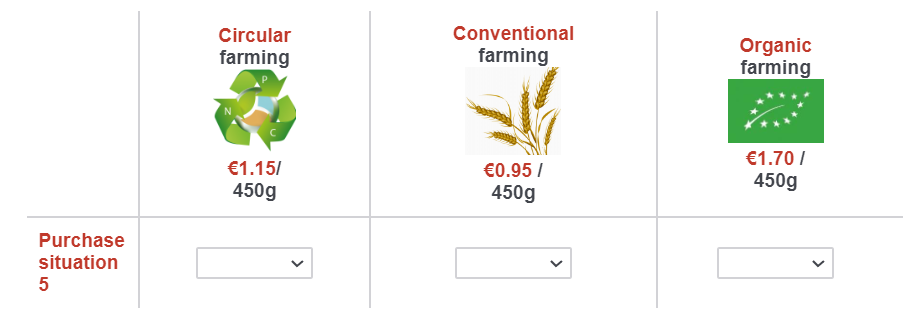


**Q2.10 THE FREQUENCY OF FOOD PURCHASES**

Q2.10.1 How often do you go for groceries? (Frequency)—Food in general

Q2.10.2 How often do you go for groceries? (Frequency)—Sliced pork loin

Q2.10.3 How often do you go for groceries? (Frequency)—Milk

Q2.10.4 How often do you go for groceries? (Frequency)—Bread

- Daily
- 2–3 times a week
- Once a week
- 2–3 times per month
- Once a month or less
- Never

**Section 3 Environmental Attitudes**

Q3.1 How much do you agree or disagree with the following statements?


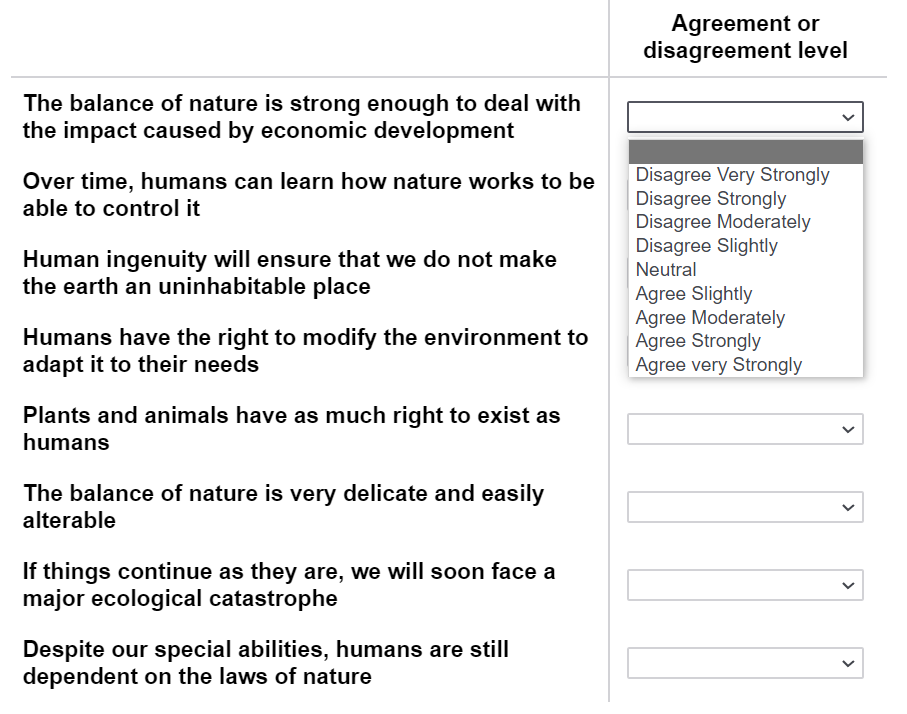


**Section 4 Opinions on the Sustainability of Different Production Systems and Dietary Patterns**

Q4.1 In your opinion, what impact do the following farming systems have on the environment?


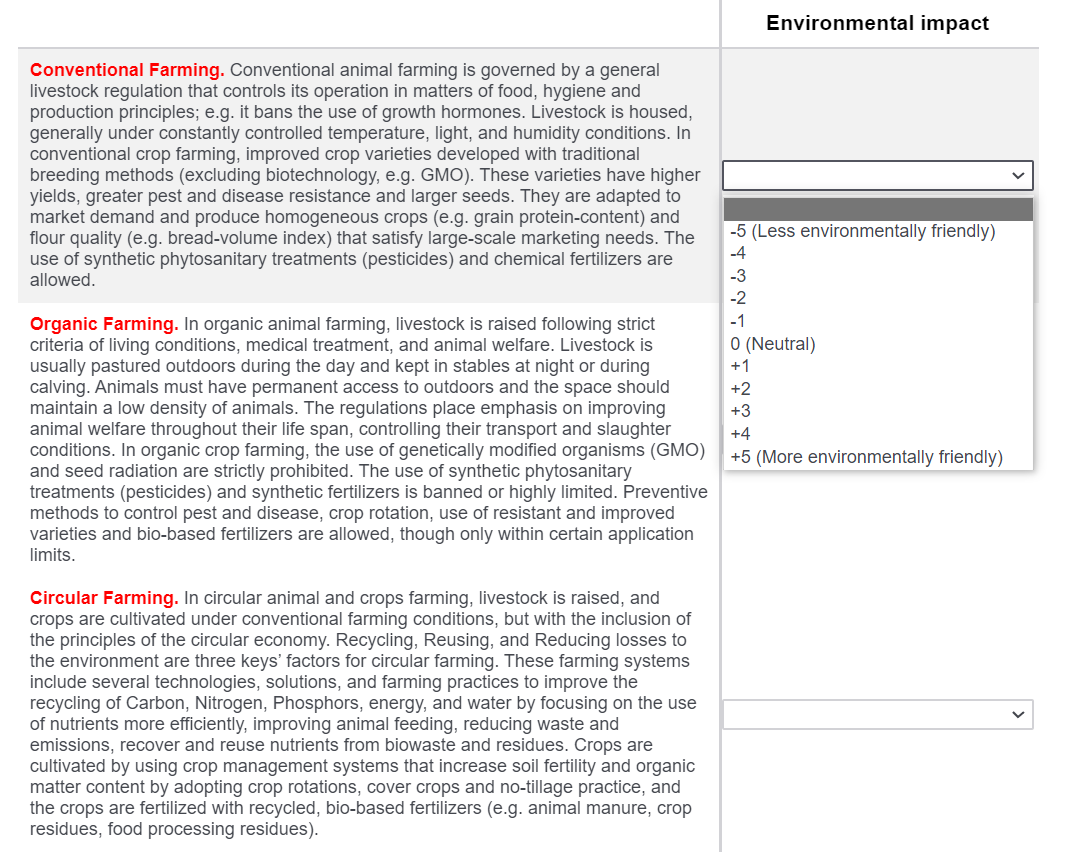


Q4.2 In your opinion, based on your understanding of what Environmental Sustainability means, **what level of environmental sustainability do the following dietary patterns have?**

| Vegetarian (It consists of not consuming meat or fish. The basis of this diet is vegetables, legumes, fruits, cereals, seeds, and nuts. It also includes the consumption of animal products such as dairy (e.g., yogurt, milk, and cheese) |  | 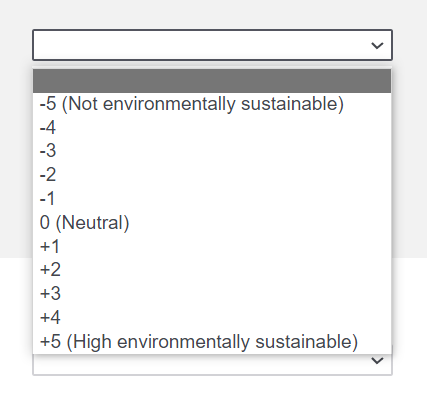 |
| --- | --- | --- |
| Vegan (It consists of not consuming any food of animal origin, such as meat, fish, eggs, or dairy) |  | 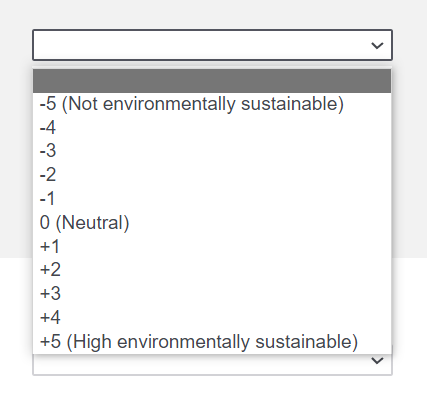 |
| Flexitarian (It consists of increasing the consumption of vegetables and fruits, and reducing the consumption of animal meats) |  | 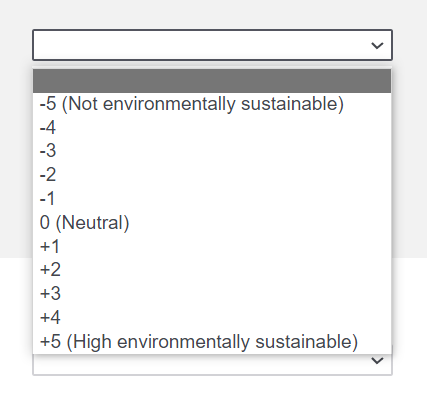 |
| Non-restricted diet (It consists of consuming any animal or non-animal food without any restriction) |  | 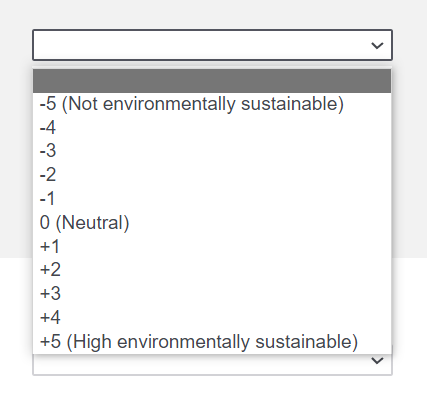 |

|  |
| --- |

The survey has ended. Thank you for your participation!
